# Development of a Multigram Synthetic Route to RM-581, an Orally Active Aminosteroid Derivative Against Several Types of Cancers

**DOI:** 10.3390/molecules30112441

**Published:** 2025-06-03

**Authors:** René Maltais, Doriane de Sainte Maresville, Vincent Desrosiers, Donald Poirier

**Affiliations:** 1 Laboratory of Medicinal Chemistry, Endocrinology and Nephrology Unit, CHU de Québec Research Center-Université Laval, Québec, QC G1V 4G2, Canada; rene.maltais@crchudequebec.ulaval.ca (R.M.); dorianedsm@hotmail.fr (D.d.S.M.); vincent.desrosiers.1@crchudequebec.ulaval.ca (V.D.); 2 Department of Molecular Medicine, Faculty of Medicine, Université Laval, Québec, QC G1V 0A6, Canada

**Keywords:** steroid, chemical synthesis, amidation, NMR, anticancer agent

## Abstract

Aminosteroid derivative RM-581 stands out as an anticancer agent, supported by positive in vitro and in vivo studies on resistant cancers of the breast, prostate, and pancreas. A synthetic route has already been developed to obtain aminosteroid RM-581 in small quantities (scale of milligrams to a few grams). However, this route has significant limitations in view of its transposition to scaling up to larger quantities to support late preclinical and clinical trials. Among the problems are the use of toxic reagents, the moderate overall yield, and the need for multiple purifications through chromatographic columns. The development of a new synthetic route has therefore been explored. Starting from commercially available estrone, 2,4-dibromo-estrone was rapidly formed, followed by the regioselective introduction of a nitro group at the C2 position and by the methylation of phenol at the C3 position. The 4-bromo-2-nitro-3-*O*-methylestrone was then reduced to 2-amino-3-*O*-methylestrone and the primary amine was used to form the piperazine ring. Once the cyclization step was carried out, the last two steps were identical to the first synthetic route previously reported, i.e., introducing an ethynyl group at the C-17α position and then adding the quinoline-proline side chain with an *N*-acylation, assisted by a peptide coupling reagent. Importantly, no purification by chromatography was necessary during the whole sequence of reactions and only a final silica gel filtration, followed by recrystallization, led to RM-581 at a very high level of purity. The structure was also fully characterized by 2D NMR analysis.

## 1. Introduction

RM-581 has emerged as a lead candidate, showing strong in vitro and in vivo cytotoxic activities in a number of cancer models [1,2,3,4], including ones with bad prognoses like pancreatic, castration-resistant prostate, and triple-negative metastatic breast cancers [5,6,7]. This aminosteroid derivative consists of a quinoline-proline-piperazine chain linked at the 2-position of a mestranol core (anisole as the A-ring). Orally active, RM-581 differs from previous aminosteroids having the same chain at the 2-position but has a 5α-androstane core (3α-hydroxycyclohexane as the A-ring), such as orally inactive RM-133 [8]. Among steroids [9,10,11,12], aminosteroid derivatives are a new family of anticancer agents that trigger endoplasmic reticulum stress (ERS)-induced apoptosis [3,4], and targeting ERS represents a novel promising approach to treat solid tumors [13]. The arrival of such a new drug acting through a different mechanism of action and demonstrating efficacy against resistant cancers would be highly beneficial for patients [14,15,16].

**Scheme 1:**
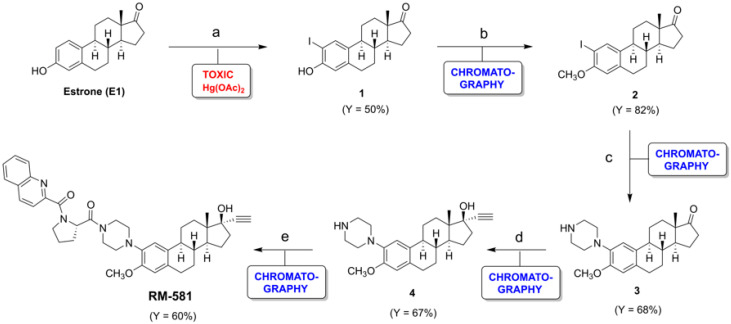
First reported chemical synthetic route to RM-581 performed in a sub-gram quantity. Problematic steps toward scale-up are highlighted in red (toxic reagent) or in blue (chromatography required). Reagents and conditions: (a) Hg(OAc)_2_, I_2_, AcOH/THF; (b) MeI, Cs_2_CO_3_, acetonitrile, reflux, 2.5 h; (c) piperazine, K_2_CO_3_, CuI, *L*-proline, DMSO, 120 °C, overnight; (d) (i) TMS-acetylene, MeLi, THF, rt, overnight; (ii) K_2_CO_3_, MeOH, rt, 5 h; (e) 1-(quinolin-2-ylcarbonyl)-*L*-proline TFA salt, HBTU, DIPEA, DMF, rt, overnight. Overall yield = 11% from E1.

A key step toward the translation of RM-581 to late preclinical studies is now to develop a reliable scale-up synthetic route for its preparation in larger quantities. In previous work, we reported the synthesis of RM-581 through a five-step convergent synthesis (Scheme 1) [17]. This short chemical route, however, was not optimal for its transposition to scaled up synthesis and had important hurdles, including: (1) the use of toxic mercury(II) acetate in the preparation of 2-iodo-estrone from estrone; (2) the variable yields, long reaction time, and large piperazine excess needed for the Ullman *N*-arylation; (3) the moderate yield for the final coupling of the quinoline side chain; and (4) the need for multiple chromatographic purifications along the sequence (4 purifications in 5 steps). Overall, this first synthetic route provided RM-581 in a global yield of 11% within five convergent steps on a 500 mg scale. Herein, we report our efforts toward an improved synthesis of RM-581 to enable its preparation in larger high-purity quantities and in a crystalline form.

## 2. Results and Discussion

### 2.1. Efficient New Route to RM-581

**Scheme 2:**
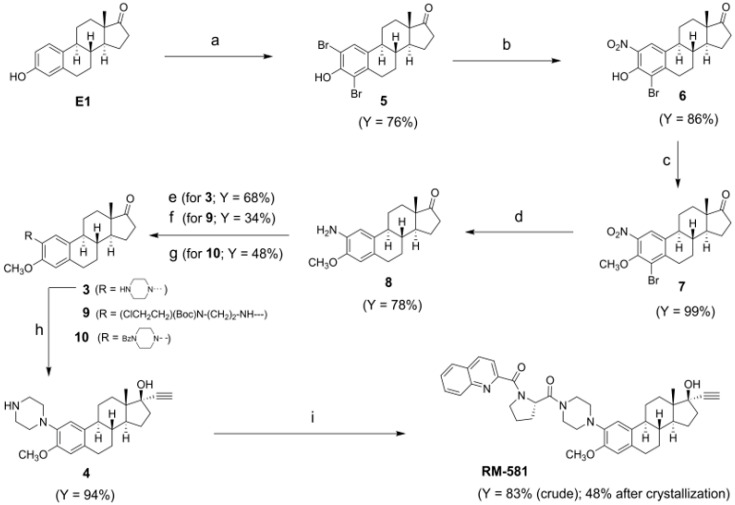
New synthetic route to multigram quantities of RM-581. Reagents and conditions: (a) NBS, DCM, 0 °C to rt, 24 h; (b) aq. NaNO_2_ (10%), AcOH, rt, 30 min; (c) CH_3_I, Cs_2_CO_3_, acetonitrile, reflux, 2 days; (d) Pd(OH)_2_ 20% on charcoal, H_2_, MeOH/DCM (85:15), rt, 12 h; (e) bis(2-chloroethyl)amine-HCl (1.05 eq.), 1-butanol, 135 °C, 4 days; (f) *Tert*-butyl bis-(2-choloroethyl) carbamate (1.4 eq.), 1-butanol, K_2_CO_3_, MW 150 °C, 12 h; (g) *N*-benzyl-bis-(2-chloroethyl)amine-HCl, 1-butanol, MW 150 °C, 8 h; (h) acetylide lithum diethylamine complex, DMSO, rt, 24 h; (i) 1-(quinolin-2-ylcarbonyl)-*L*-proline TFA salt [19], COMU, DIPEA, DMF, rt, 1 h. Overall yield = 15% from E1 (7 steps). HPLC purity of RM-581 = 99.6%.

To provide a more convenient route for the preparation of steroid derivative RM-581 on a larger scale, we tested a new strategy to replace the first steps in its previously published synthesis (Scheme 1), which were identified as the most problematic ones. Notably, the usage of toxic Hg(OAc)_2_ to introduce 2-iodo (step 1) and the large excess of piperazine and long heating time needed for the addition of piperazine through a CuI-catalyzed Ullmann amine cross-coupling reaction, giving variable yields of 30–68% (step 3), were found to be unsuitable. We thus explored the possibility of forming the piperazine ring from cyclization of aniline derivative **8** with bis-(2-chloroethyl)amine-HCl reagent (Scheme 2). This synthetic route was judged to be potentially very advantageous considering that the synthesis of 2-amino-estrone was previously reported in four steps from estrone in high yields and, most importantly, without the need for chromatographic purification [18]. This later sequence of reactions was then adapted to provide the 2-amino-3-*O*-methylestrone (**8**) required for the formation of 2-piperazine derivative **3**. Indeed, we first methylated estrone intermediate **6** using methyl iodide assisted by cesium carbonate in refluxing acetonitrile to provide 3-*O*-methylestrone intermediate **7** in a quantitative yield. The subsequent dual reduction of the 2-nitro and 4-bromo substituents under catalytic palladium hydrogenation readily provided aniline derivative **8.** As anticipated, these two later steps proceeded in high yields without the need for chromatographic purification.

We then explored different reaction conditions for the formation of piperazine derivative **3** from aniline intermediate **8** (Table 1). The use of 1-butanol as a solvent gave the most satisfactory results, and acceptable yields of **3** were only obtained using 1.05 eq. of bis-(2-chloroethyl)amine-HCl. In fact, when an excess of this reagent was used (4.0 eq.), an immediate chromatographic purification was required to remove the excess reagent and to limit intermolecular side reactions, which occurred when the compound was concentrated by evaporation, leading to a mixture of side products, with a moderate reaction yield (entry 1). To avoid the need for chromatographic purification and side reactions, we explored working at an equimolar quantity (1.05 eq.) of the reagent and extending the reaction time until observation of complete consumption of the starting material. This strategy was found to be successful, leading to an isolated yield of 68% after 4 days of heating at 135 °C (entry 2). Variation of the solvent from ethylene glycol (entry 3) gave lower yields, while no compound **3** was isolated when DMSO was used (entries 4 and 5).

In parallel with the use of the bis-(2-chloroethyl)amine-HCl side chain, we also tested the same chlorinated chain with the amine function protected by a *tert*-butyloxycarbonyl (Boc). Named *tert*-butyl bis-(2-chloroethyl) carbamate, this commercial product was added to compound **8** and heated under microwave irradiation at 150 °C for 12 h with potassium carbonate as the base (entry 6, Table 1). Unfortunately, at the end of the microwave irradiation, the sole product isolated after chromatographic purification was the mono-addition of the carbamate chain on the amino group of compound **8** without cyclization, providing **9** in a low 34% yield. In another strategy, the reaction of *N*-benzyl protected bis-(2-chloroethylamine)-HCl with compound **8** and microwave heating in 1-butanol for 8 h provided 48% yield of the corresponding *N*-benzylated cyclization compound **10** (entry 7). However, considering that the use of microwave heating is not compatible with scale-up purposes, the need for a chromatographic purification, the lower yield, and the need of a subsequent debenzylation step to obtain the free amine (compound **3**), the unprotected bis-(2-chloroethyl)amine-HCl reagent and the reaction conditions of the entry 2 (Table 1) were chosen to progress with the rest of the synthetic sequence leading to RM-581.

The crude compound **3** obtained with the optimized cyclization reaction conditions of entry 2 was then reacted with lithium acetylide-ethylenediamine complex in DMSO at room temperature for 24 h to provide compound **4** in quantitative yield, without the need for chromatographic purification (Scheme 2). This methodology of ethynylation was more advantageous than the previous two-step procedure using lithium trimethylsilyl-acetylene followed by basic hydrolysis [17], where a chromatographic purification was necessary to prevent the formation of *N*-trimethylsilyl side product.

The last step in the new synthesis of RM-581, the installation of the 1-(quinolin-2-ylcarbonyl)-l-proline side chain, was investigated using different coupling agents for amidation, including HBTU, T3P (propylphosphonic anhydride) [20], and COMU [21,22] (Table 2). Since the coupling yields were found to be comparable in a comparative assay using HBTU and COMU (80% vs. 83%), but lower for T3P (64%), we ultimately decided to select COMU as the most appropriate reagent, since it was reported to provide by-products that can be easily removed by washing with water, unlike HBTU. The use of COMU would thus facilitate the synthesis of larger quantities by eliminating the need for chromatographic purification. Following the selection of COMU, we then proceeded to the coupling of intermediate **4** with the activated ester of the quinoline side chain (1-quinolin-2-ylcarbonyl-*L*-proline TFA salt), leading to multigram quantities of RM-581. This final compound was then obtained in pure form by recrystallization (99.6% HPLC) and obtained in acceptable yield (48%).

### 2.2. NMR Characterization of RM-581

The NMR signals of second-generation aminosteroids with a C18-steroid nucleus, such as RM-581, unlike first-generation aminosteroids with a C19-steroid nucleus, such as RM-133 [8], have never been fully assigned, despite the presence of two different backbones. HSQC, HMBC, COSY, and NOESY 2D NMR experiments and some published data were thus used to first perform the carbon assignment and, subsequently, to complete the proton assignment (Table S1 and Figures S2–S19; Supplementary Materials). It was important to perform a careful assignment, since the presence of the quinoline-proline-piperazine chain at C2 induces constraints that cause most of the ^1^H (Figure 1) and ^13^C (Figure 2) NMR signals to split.

Starting with the steroid nucleus, the carbon and proton signals of the B-, C-, and D-rings were readily identified using data reported in the literature [19], but not the signals of the A-ring, which underwent proton splitting, being located near the C2 side chain. It is also worth mentioning that, despite their opposite position (D-ring) to the side chain attached to the A-ring, the three Hs and Cs of CH_3_-18 underwent small splitting (0.90/0.93 ppm and 12.39/12.44 ppm), as did acetylenic CH-21 (2.97/3.02 ppm and 73.6/73.7 ppm). Similar effects were also observed for C17 (78.7/78.9 ppm) in ^13^C NMR. For the A-ring, C4 and C1 were first identified through HMBC (^3^*J*_HCCC_) and NOESY correlations with CH_2_-6 and CH-9, respectively. The HSQC (^1^*J*_HC_) correlations then allowed the identification of H4 (6.58 and 6.66 ppm, 2 singlets) and H1 (6.55 and 6.90 ppm, 2 singlets), the latter being clearly more affected by the proximity of the C2 side chain. Similarly, the CH_3_O singlet was split (3.75 and 3.85 ppm) in ^1^H NMR, while the effect was much weaker in ^13^C NMR (54.9 and 55.0 ppm). The HMBC correlations next allowed the identification of C2, C3, C5, and C10. Briefly, C3 was identified by a ^3^*J*_HCCC_ correlation with CH_3_O, C5 was the only aryl carbon to show a ^3^*J*_HCCC_ correlation with H7, C10 provided a ^2^*J*_HCC_ correlation with H9, and C2 was the only aryl carbon that showed ^2^*J*_HCC_ and ^3^*J*_HCCC_ correlations with H1 and H4, respectively.

**Figure 3:**
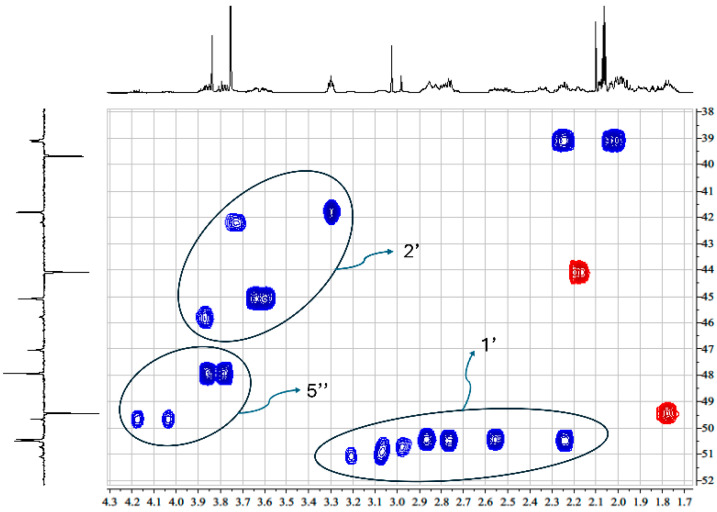
Partial HSQC NMR spectrum (1.5 to 4.3 ppm and 37 to 53 ppm) illustrating the complexity of ^1^H and ^13^C NMR signals associated with CH_2_-1′, 2′, and 5″.

The aromatic nucleus of the steroidal moiety did not significantly affect the ^1^H and ^13^C NMR chemical shifts of the left-hand portion (quinoline-proline) of the side chain, which facilitated the identification of both C and H. In fact, the HMBC, HSQC, and NOESY correlations obtained for RM-581 well supported those already identified for the side chain when it was attached at C2 of a C19-steroid derivative (RM-133) [19]. To illustrate these significant splittings, especially in ^1^H NMR, we retained the signals of the methine (CH-2″) of the proline, which were found at 5.20 and 5.90 ppm (^1^H NMR) and 57.6 and 59.2 ppm (^13^C NMR). Thanks to the HMBC (^3^*J*_HCCC_ and ^2^*J*_HCC_) correlations, these two very characteristic signals allowed the identification of the three methylenes (CH_2_-3″, 4″, and 5″) of the proline, as well as the C-1″ carbonyl. The latter also showed a ^3^*J*_HCCC_ correlation with the two H2-2′ signals of piperazine in the HMBC spectrum. In the COSY spectrum, correlations with H2-2′ allowed the localization of the signals for H2-1′. As expected, the H of CH_2_-1′ (H of an amine) was less deshielded than the H of CH_2_-2′ (H of an amide). Positioned between proline and the aromatic A-ring of the steroid, the CH_2_-1′ and CH_2_-2′ signals of the piperazine nucleus were very affected, with 4 signals for each of the two CH_2_ in ^13^C NMR as well as several signals in ^1^H NMR (Table S1; Supplementary Materials). As reported in Figure 3, the HSQC NMR spectrum of RM-581 clearly illustrates the complex splitting of CH_2_-1′ and CH_2_-2′ piperazine signals and CH-5″ proline signals associated with the two forms of RM-581.

As mentioned above, the NMR analysis of RM-581 showed the presence of two forms with different chemical shifts. Previous analyses carried out with C19-steroid derivative RM-133 dissolved in four deuterated solvents also showed the impact of the solvent on the proportion of the two forms [19]. Thus, for the ^1^H NMR analysis of RM-581 in acetone-d_6_, the proportion was determined as 73:27 when assessed with the integration of seven well-defined and characteristic signals (1, 4, 2″, 3‴, 4‴, 8‴, and 9‴), and similar proportions were obtained with RM-133 in the same deuterated solvent. Splitting was also observed for the same ^13^C NMR signals. In fact, splitting occurred with the addition of the proline moiety and the presence of two amide bonds, inducing a rotation restriction and the formation of two conformers. So, the use of an estrane (C18-steroid) instead of an androstane (C19-steroid) nucleus therefore had no impact on the side chain arrangement and the proportion of its two forms.

## 3. Materials and Methods

### 3.1. General

Chemical reagents and anhydrous dimethylformamide (DMF) were purchased from Sigma-Aldrich Canada Ltd. (Oakville, ON, Canada). 1-Cyano-2-ethoxy-2-oxoethylidenaminooxy) dimethylamino-morpholino-carbenium hexafluorophosphate (COMU) and hexafluorophosphate benzotriazole tetramethyl uranium (HBTU) were purchased from Matrix Innovation (Québec, QC, Canada). The usual solvents were obtained from Fisher Scientific (Montréal, QC, Canada) and were used as received. Thin-layer chromatography (TLC) and flash column chromatography were performed on 0.20 mm silica gel 60 F254 plates (Merck, Darmstadt, Germany) and with 230–400 mesh ASTM silica gel 60 (Silicycle, Québec, QC, Canada), respectively. Nuclear magnetic resonance (NMR) spectra were recorded at 300 MHz for ^1^H and 75 MHz for ^13^C on a Bruker Avance NEO 300 digital spectrometer (Billerica, MA, USA) or at 500 MHz for ^1^H and 125 MHz for ^13^C on an Agilent Direct-Drive (DD2) spectrometer (Agilent Technologies, Santa Clara, CA, USA). The chemical shifts (δ) were expressed in ppm and referenced to chloroform-d (7.28 and 77.0 ppm, acetone-d_6_ (2.05 and 28.9 ppm) for ^1^H and ^13^C NMR, respectively. High-performance liquid chromatography (HPLC) analyses for chemical purity were performed on a Shimadzu Prominence instrument (Kyoto, Japan) using a diode array detector and an Altima C18 analytical reverse phase column (5 µm, 4.6 × 250 mm) applying the conditions stated (wavelength detection and solvent gradient). Low-resolution mass spectra (LRMS) were recorded on a Shimadzu Prominence instrument (Kyoto, Japan) equipped with a Shimadzu LCMS-2020 mass spectrometer and an atmospheric pressure chemical ionization (APCI) probe and are expressed in *m*/*z*.

### 3.2. Chemical Synthesis of RM-581

#### 3.2.1. Synthesis of 2,4-Dibromo-3-hydroxyestra-1(10),2,4-trien-17-one (**5**)

To a solution of estrone (100 g, 370 mmol) in dichloromethane (DCM) (1.5 L) at 0 °C under an argon atmosphere was slowly added *N*-bromosuccinimide (NBS) (198 g, 1112 mmol). The solution was allowed to return to room temperature and stirred for 24 h. A 10% NH_4_Cl aqueous solution was then slowly added at 0 °C and the mixture was stirred for 15 min. The resulting solution was washed with water (3 × 1 L), and dried over anhydrous MgSO_4_, filtered, and evaporated under reduced pressure. The crude product was then dissolved in DCM (65 mL), methanol (MeOH) was added (250 mL), and the mixture stirred for 15 min. The resulting solution was dried over anhydrous Na_2_SO_4_, filtered, and evaporated under reduced pressure to give compound **5** (120.5 g, 76% crude yield). The ^1^H NMR data were in full agreement with those reported in the literature [23].

#### 3.2.2. Synthesis of 4-Bromo-3-hydroxy-2-nitroestra-1(10),2,4-trien-17-one (**6**)

Compound **5** (120 g, 280 mmol) was dissolved in glacial acetic acid (2.5 L) at room temperature under an argon atmosphere. Aqueous sodium nitrite (10%) (38.4 mL) was then slowly added, and the resulting solution was stirred for 24 h. The acetic acid was evaporated under reduced pressure. The crude compound was dissolved in ethyl acetate (EtOAc) (1 L), poured into NaOH (2.0 M) solution (2 L) on ice, and washed. The organic layer was successively washed with a 10% NH_4_Cl aqueous solution and brine, dried over anhydrous Na_2_SO_4_, filtered, and evaporated under reduced pressure. The resulting compound was triturated with EtOAc and filtered to give compound **6** (95.4 g, 86% crude yield). The NMR data were in full agreement with those reported in the literature [18].

#### 3.2.3. Synthesis of 4-Bromo-3-methoxy-2-nitroestra-1(10),2,4-trien-17-one (**7**)

To a stirred solution of compound **6** (95.4 g, 241 mmol) in acetonitrile (2 L) was added cesium carbonate (158 g, 485 mmol) and methyl iodide (204 g, 90 mL, 1438 mmol). After refluxing for 24 h, the resulting solution was poured into water and extracted with EtOAc. The organic layer was washed with brine, dried with anhydrous Na_2_SO_4_, filtered, and evaporated under reduced pressure to give compound **7** (99.0 g, 99% crude yield). HPLC purity = 98.7%. ^1^H NMR (300 MHz, CDCl_3_, δ in ppm): 0.93 (s, 3H, CH_3_-18), 1.41–1.79 (m, 6H, CH and CH_2_ residual), 1.97–2.49 (m, 6H, CH et CH_2_ residual), 2.55 (dd, 1H, *J* = 18.0 Hz, *J* = 9.0 Hz, 16β-CH), 2.77–3.18 (m, 2H, CH_2_-6), 4.00 (s, 3H, OCH_3_), 7.81 (s, 1H, CH-1). ^13^C NMR (75 MHz, CDCl_3_, δ in ppm): 13.7, 21.5, 25.9, 26.3, 29.7, 31.6, 35.8, 37.0, 44.1, 47.7, 50.2, 62.4, 121.1, 123.1, 138.2, 142.3, 143.9, 148.8, 221.1. LRMS for C_19_H_23_NO_4_^81^Br [M + H]^+^ 409.8 *m*/*z*.

#### 3.2.4. Synthesis of 2-Amino-3-methoxy-estra-1(10),2,4-trien-17-one (**8**)

Compound **7** (24.5 g, 60.0 mmol) was dissolved in a mixture of MeOH (1 L) and DCM (150 mL) under an argon atmosphere. Pd(OH)_2_ (20% on charcoal, 50% wet) (4.5 g) was added, and the mixture purged three times with H_2_ and then stirred overnight. Pd(OH)_2_ was then filtered through Celite, and the MeOH was evaporated before adding saturated NaHCO_3_ aqueous solution. After extraction with EtOAc, the combined organic phase was washed with brine, dried over anhydrous Na_2_SO_4_, filtered, and evaporated under reduced pressure. The crude compound was dissolved in EtOAc (1 L) and MeOH (200 mL), and the organic layer was washed with 10% HCl aqueous solution. The resulting aqueous phase was washed with EtOAc and then neutralized with 10% NaHCO_3_ aqueous solution. The aqueous phase was extracted with EtOAc, washed with brine, dried with anhydrous Na_2_SO_4_, filtered, and evaporated under reduced pressure to give compound **8** as an amorphous brown solid (14.0 g, 78% crude yield). The reaction was repeated four times on the same scale to obtain a total of 56 g. HPLC purity = 90.2%. ^1^H NMR (300 MHz, CDCl_3_, δ in ppm): 0.93 (s, 3H, CH_3_-18), 1.35–1.73 (m, 6H, CH and CH_2_ residual), 1.90–2.40 (m, 6H, CH and CH_2_ residual), 2.51 (dd, 1H, *J* = 18.0 Hz, *J* = 9.0 Hz, 16β-CH), 2.83–2.88 (m, 2H, CH_2_-6), 3.69 (s, 2H, NH_2_), 3.84 (s, 3H, OCH_3_), 6.55 (s, 1H, CH-4), 6.70 (s, 1H, CH-1). ^13^C NMR (75 MHz, CDCl_3_, δ in ppm): 13.8, 21.5, 26.0, 26.8, 29.1, 31.6, 35.8, 38.4, 44.0, 48.0, 50.4, 55.5, 111.0, 112.2, 126.2, 131.8, 133.8, 145.7, 221.0. LRMS for C_19_H_26_NO_2_ [M + H]^+^ 300.1 *m*/*z*.

#### 3.2.5. Synthesis of 3-Methoxy-2-piperazin-1-yl-estra-1(10),2,4-trien-17-one (**3**) 

To a solution of compound **8** (55.8 g, 186.4 mmol) in 1-butanol (300 mL) and under an argon atmosphere was added bis-(2-chloroethyl)amine hydrochloride (33.3 g, 186.5 mmol), and the mixture was heated at 135 °C for 4 days. The resulting solution was cooled and poured into water (3 L). The aqueous phase was extracted with EtOAc (3 × 250 mL), then neutralized with 10% NaHCO_3_ aqueous solution, and extracted with EtOAc (3 × 500 mL). The combined organic layer was washed with brine, dried with anhydrous Na_2_SO_4_, filtered, and evaporated under reduced pressure. The crude compound was co-evaporated with H_2_O/MeOH to remove the residual 1-butanol, leading to 46.5 g (68% crude yield) of compound **3**. HPLC purity = 97.9%. The NMR data were in full agreement with those reported in the literature [17].

#### 3.2.6. Synthesis of 17α-Ethynyl-3-methoxy-2-piperazine-estra-1(10),2,4-trien-17β-ol (**4**)

To compound **3** (46.4 g, 126.0 mmol) in anhydrous dimethylsulfoxide (DMSO) (1 L) was successively added, at 60 min intervals, three portions of lithium acetylide-ethylenediamine complex (46.4 g, 503.8 mmol). The resulting mixture was stirred for 24 h at room temperature and then slowly poured into cold water (3 L). The aqueous phase was extracted with EtOAc, washed with brine, dried with anhydrous Na_2_SO_4_, filtered, and evaporated under reduced pressure to give 46.7 g (94% crude yield) of compound **4**. HPLC purity = 96.9%. The NMR data were in full agreement with those reported in the literature [17].

#### 3.2.7. Synthesis of {4-[17α-Ethynyl-17β-hydroxy-3-methoxyestra-1(10)2,4-trien-2-yl]piperazin-1-yl}[(2*S*)-1-(quinolin-2-ylcarbonyl)pyrrolidin-2-yl]-methanone (RM-581)

The 1-quinolin-2-ylcarbonyl-L-proline-TFA salt (13.8 g, 35.9 mmol) [19] and COMU (15.4 g, 35.9 mmol) were dissolved in anhydrous DMF (150 mL) under an inert atmosphere. Diisopropylethylamine (DIPEA) (15.5 mL, 89.7 mmol) was added and the mixture was stirred for 15 min. A solution of compound **4** (11.8 g, 29.9 mmol) in anhydrous DMF (50 mL) was then added, and the reaction mixture was stirred at room temperature for 2 h. The reaction was quenched with four volumes of saturated NH_4_Cl aqueous solution, and the product was recovered by filtration and air-dried under vacuum. The resulting solid was dissolved in acetonitrile and evaporated to dryness. The solid was then recrystallized. The filtrate was recovered, concentrated, and further purified by filtration on silica gel, using hexane:acetone (1:1) as the eluent. The obtained solid was recrystallized again. Overall, the title compound (RM-581) was obtained as pale-yellow needles (8.49 g, 48% yield). Rf = 0.15 by TLC in hexane:acetone (1:1). HPLC purity = 99.6% (Figure S1). The LRMS and NMR data were in full agreement with those reported in the literature [17], and a full assignment of all carbons and protons is provided in Table S1 and Figures S2–S19 (Supplementary Materials).

## 4. Conclusions

This work has enabled the development of a chemical synthetic route leading to multigram quantities of steroid derivative RM-581. Advantageously, this new convergent synthetic route of 7 steps allowed for the preparation of 8.5 g of RM-581 from commercially available estrone in a 15% overall yield. Notably, this chemical synthesis proceeds without the need for chromatographic purification, and RM-581 was obtained in crystalline form with high purity. This new synthetic route will enable the preparation of multigram quantities required to support late preclinical studies and will also be helpful in the design of a larger scale synthetic route necessary for clinical trials.

## Figures and Tables

**Figure 1:**
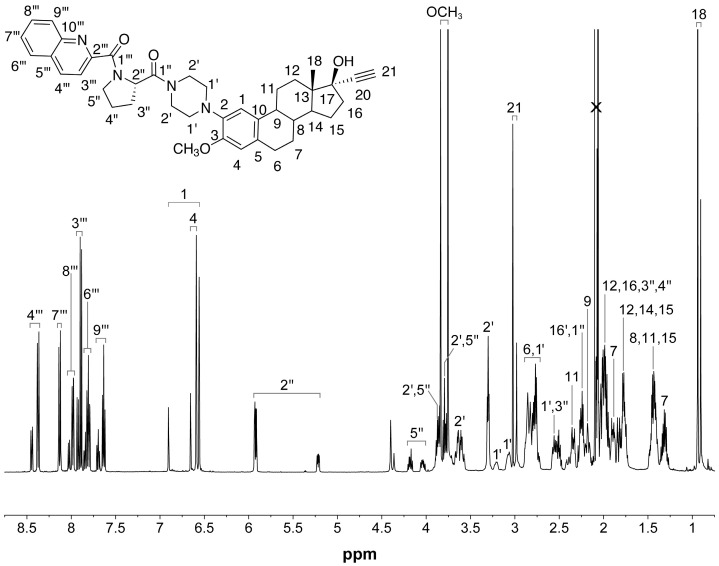
^1^H NMR spectrum of RM-581 in acetone-d_6_ showing proton assignments and signal splitting. See Scheme 2 for the configuration of carbons 8, 9, and 14.

**Figure 2:**
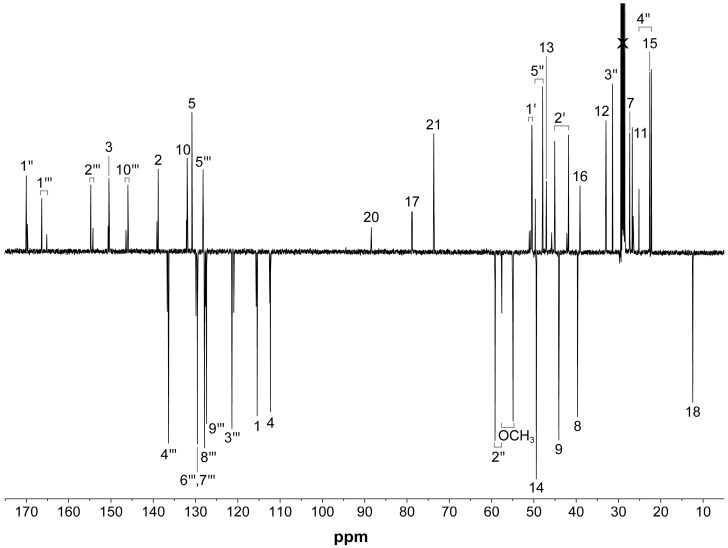
^13^C NMR spectrum of RM-581 in acetone-d_6_ showing carbon assignments and signal splitting.

**Table 1:** Optimization of cyclization providing piperazine derivative **3** from **8** (Scheme 2).

Entry	Reagent (A, B, or C) (Equivalent) ^1^	Solvent (Base)	Time (h)	Temp (°C)	Chromato. Purification	Isolated Yield (%)	HPLC Purity (%)
1	A (4.0)	1-Butanol (--)	27	150	Yes	47	23.0
2	A (1.05)	1-Butanol (--)	96	135	No	68	97.9
3	A (1.05)	Ethylene glycol (--)	27	150	No	25	88.4
4	A (1.05)	DMSO (--)	24	135	No	0 ^2^	ND ^3^
5	A (4.0)	DMSO (--)	24	135	No	0 ^2^	ND ^3^
6	B (1.4)	1-Butanol (K_2_CO_3_)	12	150	Yes	0 (34 of **9**)	ND ^3^
7	C (4.0)	1-Butanol (--)	8	150	Yes	0 (48 of **10**)	ND ^3^

^1^ Reagent A: Bis-(2-chloroethyl)amine-HCl; Reagent B: *Tert*-butyl bis-(2-choloroethyl) carbamate; Reagent C: *N*-benzyl-bis-(2-chloroethyl)amine-HCl; ^2^ Degradation, no trace of compound observed by TLC analysis. ^3^ ND: Not determined.

**Table 2:** Comparative efficiencies of coupling reagents for amidation of **4** to RM-581.

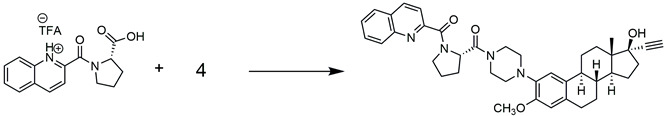			
Entry	Coupling Reagent	Equivalent	RM-581 Yield (%)
1	COMU	1.2	83
2	HBTU	1.2	80
3	T3P	2	64

Reagents and conditions: (i) Acid (1.2 eq.), coupling reagent, DIPEA (3 eq.), DMF, rt, 15 min; (ii) Compound **4** (1 eq.), DMF, rt, 2 h.

## Data Availability

Data are contained within the article and Supplementary Materials.

## Supplementary Materials

The following supporting information can be downloaded at: https://www.mdpi.com/article/10.3390/molecules30112441/s1, Figure S1: HPLC chromatogram of crystallized RM-581; Table S1: ^13^C and ^1^H NMR signal assignments for RM-581 in acetone-d_6_; Figures S2–S19: ^1^H NMR, ^13^C NMR, HSQC, HMBC, COSY, and NOESY spectra of RM-581 in acetone-d_6_.
