# Supplementary material for: Development of a Multigram Synthetic Route to RM-581, an Orally Active Aminosteroid Derivative Against Several Types of Cancers
Source: Molecules. 2025 Jun 3;30(11):2441. doi: 10.3390/molecules30112441 (PMC12156004; doi:10.3390/molecules30112441)

Supplementary Materials

Table of Contents

1. Figure S1. HPLC chromatogram of crystallized RM-581
2. Table S1. ^{13}C and ^{1}H NMR signal assignments for RM-581 in acetone-d_6
3. Figures S2A,B. ^{1}H NMR spectrum of RM-581 in acetone-d_6 (500 MHz); 0 to 10 ppm
4. Figures S3A,B. ^{13}C NMR spectrum of RM-581 in acetone-d_6 (125 MHz); 0 to 200 ppm
5. Figure S4. ^{13}C NMR spectrum of RM-581 in acetone-d_6 (125 MHz); 0 to 60 ppm
6. Figure S5. ^{13}C NMR spectrum of RM-581 in acetone-d_6 (125 MHz); 60 to 100 ppm
7. Figure S6. ^{13}C NMR spectrum of RM-581 in acetone-d_6 (125 MHz); 110 to 175 ppm
8. Figure S7. HSQC spectrum of RM-581 in acetone-d_6; 0.5 to 10 / 10 to 180 ppm
9. Figure S8. HSQC spectrum of RM-581 in acetone-d_6; 0.5 to 6.0 / 10 to 65 ppm
10. Figure S9. HSQC spectrum of RM-581 in acetone d_6; 6.3 to 8.8 / 108 to 140 ppm
11. Figure S10. HMBC spectrum of RM-581 in acetone-d_6; 0 to 8.5 / 0 to 180 ppm
12. Figure S11. HMBC spectrum of RM-581 in acetone-d_6; 0.5 to 4.5 / 10 to 90 ppm
13. Figure S12. HMBC spectrum of RM-581 in acetone-d_6; 5.6 to 8.8 / 125 to 180 ppm
14. Figure S13. COSY spectrum of RM-581 in acetone-d_6; 0.5 to 8.5 ppm / 0.5 to 8.5 ppm
15. Figure S14. COSY spectrum of RM-581 in acetone-d_6; 0.5 to 4.0 ppm / 0.5 to 4.0 ppm
16. Figure S15. COSY spectrum of RM-581 in acetone-d_6; 7.5 to 8.6 ppm / 7.5 to 8.6 ppm
17. Figure S16. NOESY spectrum of RM-581 in acetone-d_6; 0 to 9.0 ppm / 0 to 9 ppm
18. Figure S17. NOESY spectrum of RM-581 in acetone-d_6; 0 to 4.5 ppm / 0.5 to 4.5 ppm
19. Figure S18. NOESY spectrum of RM-581 in acetone-d_6; 5.8 to 7.0 ppm / 2.0 to 4.0 ppm
20. Figure S19. NOESY spectrum of RM-581 in acetone-d_6; 7.5 to 8.7 ppm / 7.5 to 8.7 ppm

FIGURE S1

==== Shimadzu LabSolutions Analysis Report ====

C:\LabSolutions\Data\Project1\EQUIPE DONALD POIRIER\Rene Maltais 2024\VD-610-099_recrist_ACN001.lcd

Acquired by	: System Administrator
Sample Name	: VD-610-099_recrist_ACN
Sample ID	: VD-610-099_recrist_ACN
Tray#	: 1
Vial#	: 1
Injection Volume	: 5
Data File	: C:\LabSolutions\Data\Project1\EQUIPE DONALD POIRIER\Rene Maltais 2024\VD-610-0
Method File	: C:\LabSolutions\Data\Project1\EQUIPE DONALD POIRIER\Rene Maltais 2022\Copy of L
Report Format File	: C:\LabSolutions\Data\Project1\EQUIPE ERIC BIRON\ERIC-2017\RAPPORT-2017\LCMS
Month-Day Acquired	: 11/22/2024
Month-Day Processed	: 11/22/2024

<Chromatogram>

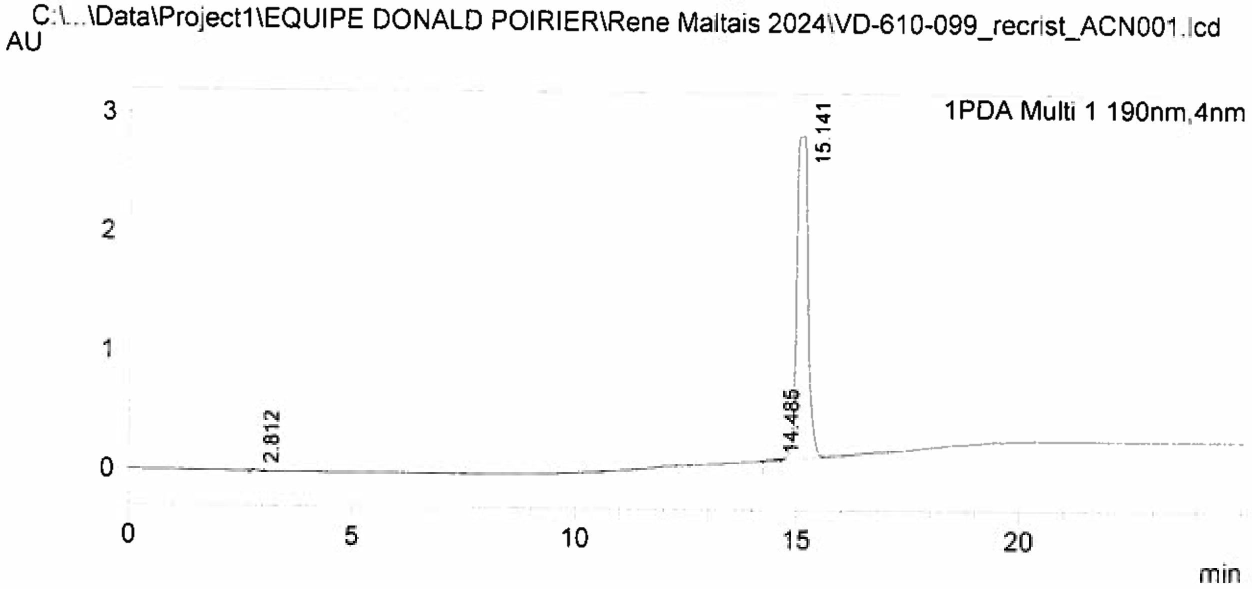

Peak Table

PDA Ch1 190nm

Peak#	Ret. Time	Area	Area%
1	2.812	83878	0.177
2	14.485	104146	0.220
3	15.141	47254991	99.604
Total		47443015	100.000

Table S1: ^{13}C NMR and ^1H NMR data for RM-581 in acetone-d_6 *

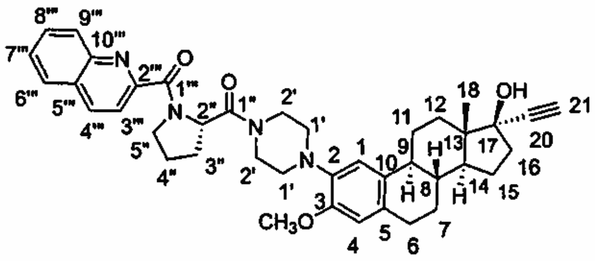

Carbon/proton Number	Multiplicity	^{13}C NMR δ in ppm	^1H NMR δ in ppm
1	CH	115.4 (115.7)	6.55 (6.90); 2s
2	C	138.8 (139.1)	--
3	C	150.4 (150.7)	--
4	CH	112.3 (112.4)	6.58 (6.66); 2s
5	C	130.9	--
6	CH_2	29.1	2.76; m
7	CH_2	27.3 (27.4)	1.31, 1.89; 2m
8	CH	39.7	1.44; m
9	CH	44.1	2.18; m
10	C	132.0 (132.1)	--
11	CH_2	26.7 (26.5)	1.44, 2.36; 2m
12	CH_2	33.0 (32.9)	1.76, 1.83, 2.00; 3m
13	C	47.1	--
14	CH	49.4	1.78; m
15	CH_2	22.6	1.44, 1.77; 2m
16	CH_2	39.1	2.03, 2.25; 2m
17	C	78.9 (78.7)	--
18	CH_3	12.44 (12.39)	0.90 (0.93); 2s
CH_3O	CH_3O	54.9 (55.0)	3.75 (3.85); 2s
20	C	88.4	--
21	CH	73.7 (73.6)	2.97 (3.02); 2s
1'	2 x CH_2	50.4 (50.7), 50.5 (51.1)	2.24, 2.55, 2.76, 2.86, 2.96, 3.06, 3.20; 7m
2'	2 x CH_2	41.8 (42.2), 45.1 (45.8)	3.30, 3.62, 3.73, 3.86 4m
1"	CO	170.0 (169.8)	--
2"	CH	59.2 (57.6)	5.20 (5.90); 2m
3"	CH_2	31.4 (28.8)	1.98, 2.00, 2.34, 2.50; 4m
4"	CH_2	22.0 (25.1)	1.96, 1.98; 2m
5"	CH_2	47.9 (49.7)	3.77, 3.85, 4.03, 4.17; 4m
1'''	CO	166.4 (165.2)	--
2'''	C	154.8 (154.2)	--
3'''	CH	121.4 (120.9)	7.89 (7.92); 2d, J = 8.7 Hz
4'''	CH	136.4 (136.7)	8.34 (8.44); 2d, J = 8.7 Hz
5'''	C	128.2 (128.3)	--
6'''	CH	129.7 (129.9)	7.81 (7.84); 2m
7'''	CH	129.6 (129.7)	8.12; d, J = 8.7 Hz
8'''	CH	127.9 (127.7)	7.97 (8.03); 2d, J = 8.7 Hz
9'''	CH	127.4 (127.8)	7.63 (7.70); 2m
10'''	C	146.0 (146.4)	--

(*) Spectra recorded at 125 MHz or 500 MHz for ^{13}C or ^1H NMR data, respectively. Data between parentheses are associated to the minor rotamer. Singlet (s), doublet (d), triplet (t) and multiplet (m). The chemical shifts were referenced to acetone centered at 28.9 ppm (^{13}C NMR) and 2.05 ppm (^1H NMR).

FIGURE S2 A

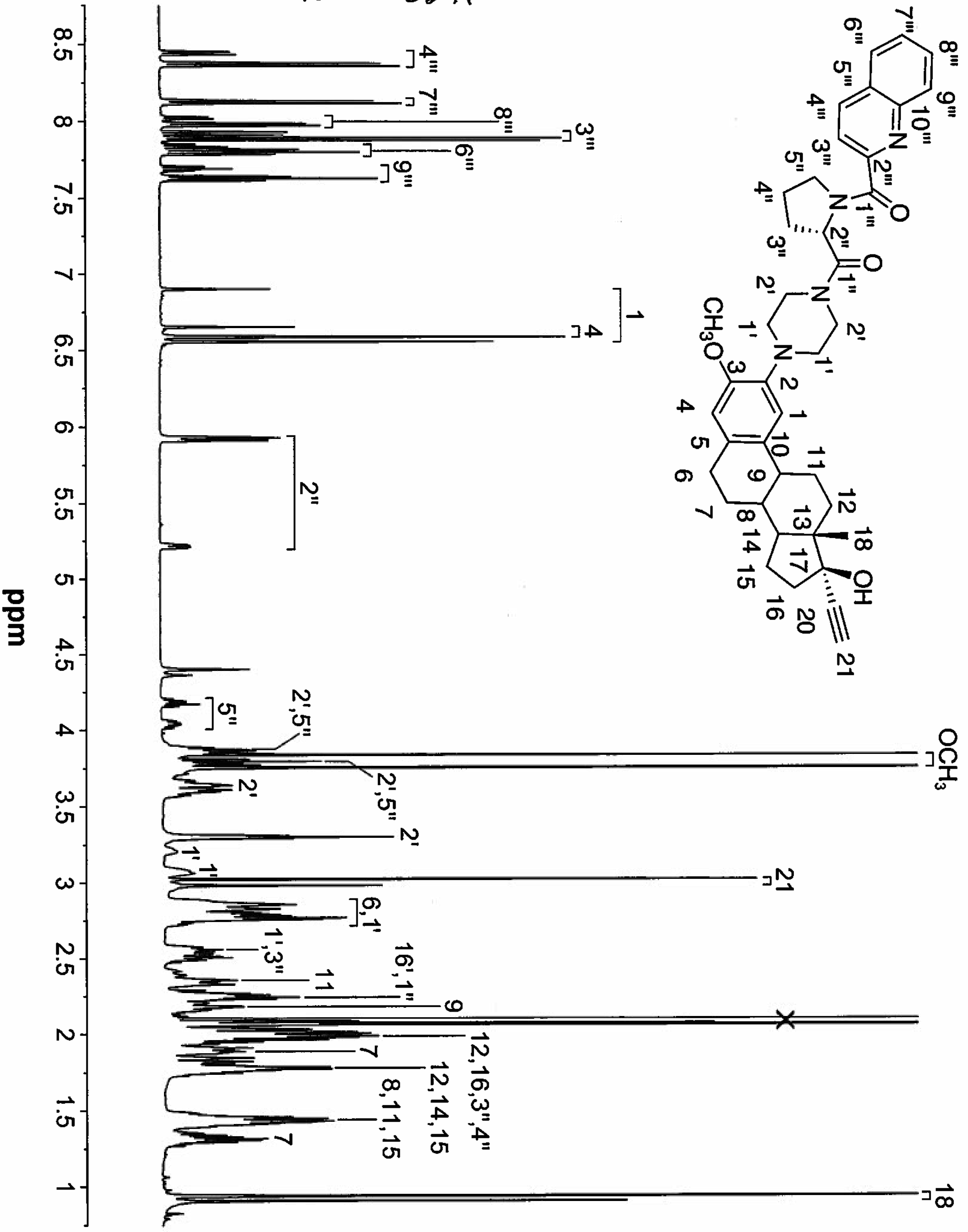

FIGURE S2B

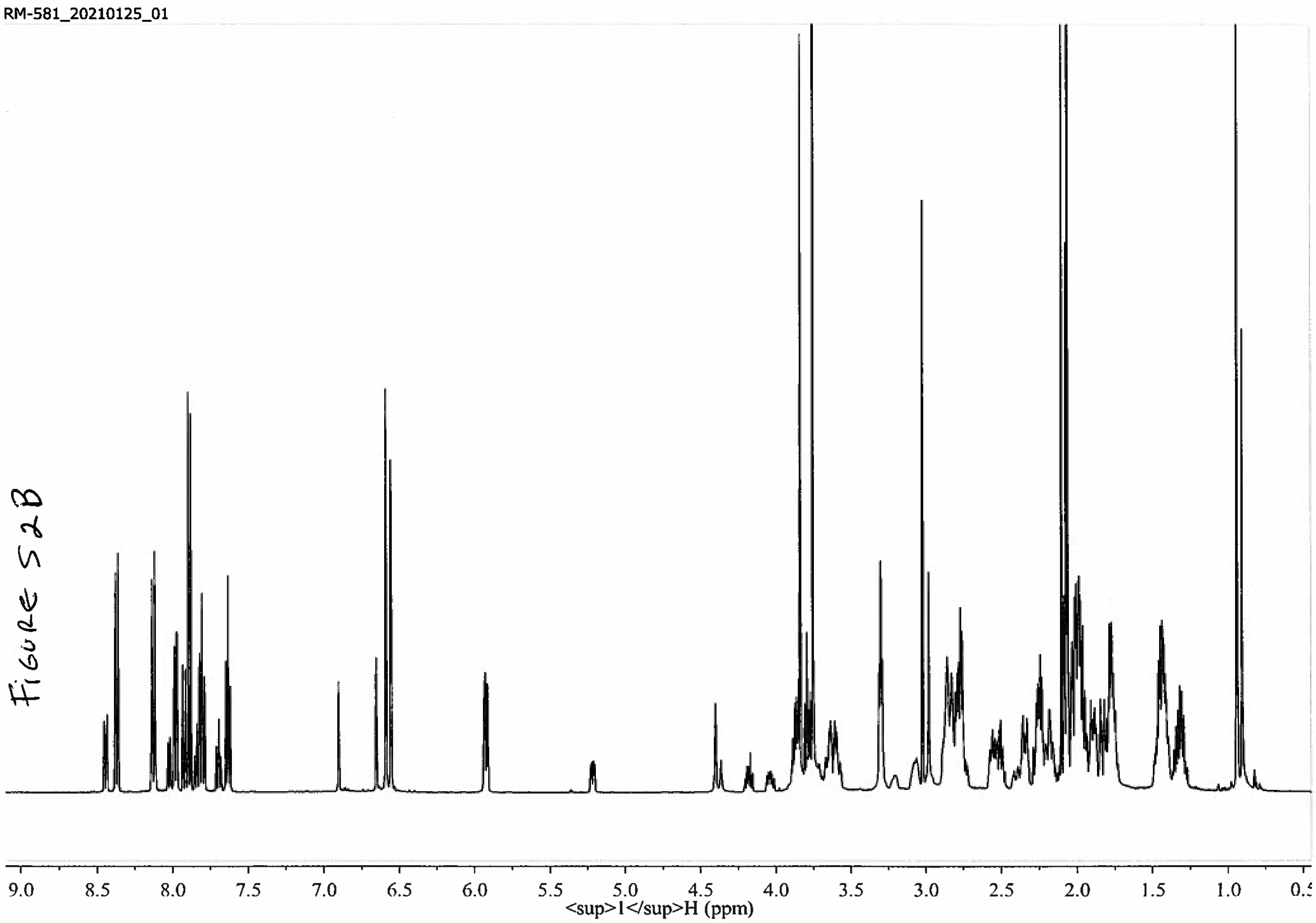
RM-581_20210125_01
9.0 8.5 8.0 7.5 7.0 6.5 6.0 5.5 5.0 4.5 4.0 3.5 3.0 2.5 2.0 1.5 1.0 0.5
¹H (ppm)

FIGURE S3A

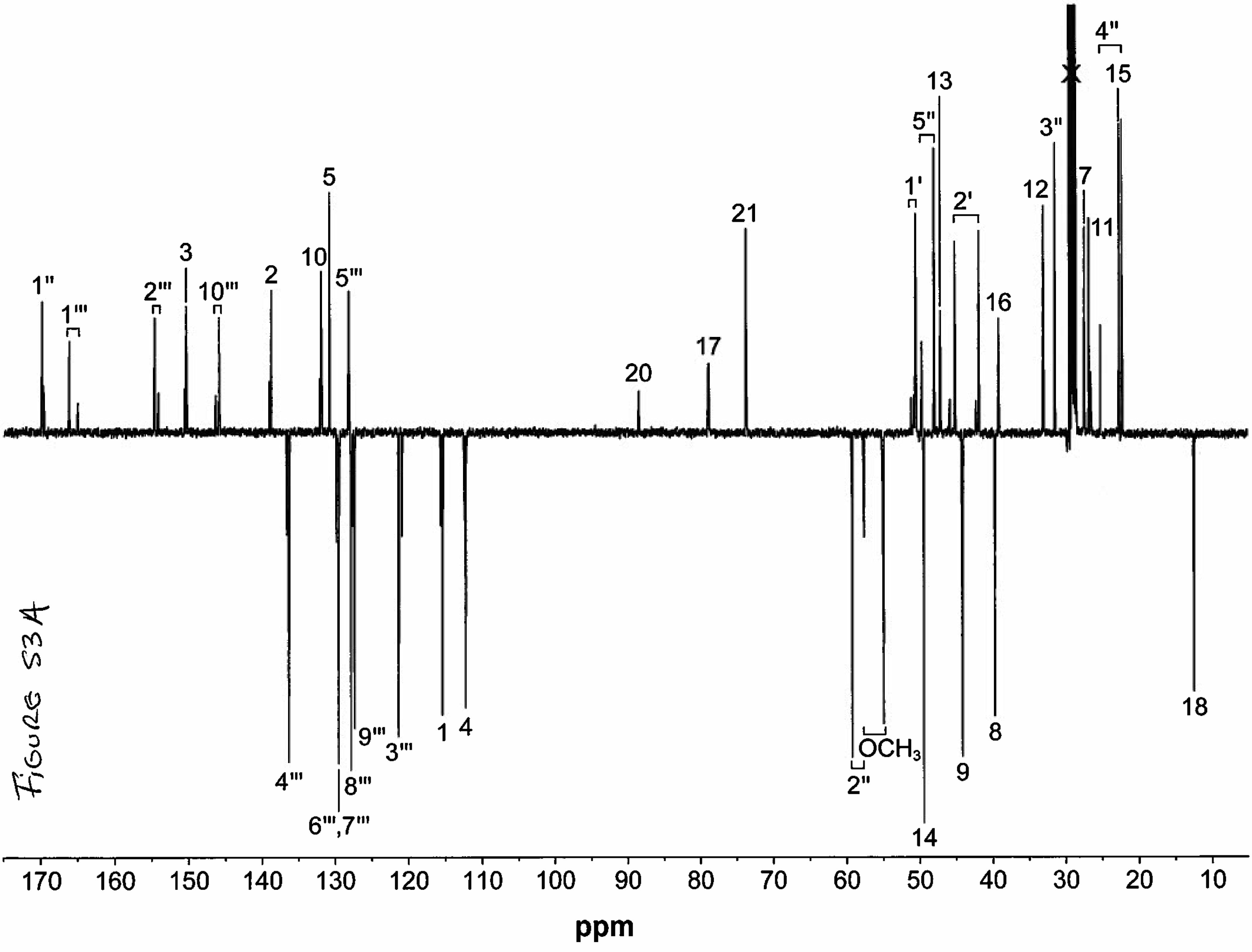

1"
1'''
2'''
3
10'''
2
10
5
5'''
20
17
21
1'
5"
13
2'
16
12
3"
7
11
4"
15
4'''
6''',7'''
8'''
9'''
3'''
1
4
2"
OCH3
14
9
8
18
170 160 150 140 130 120 110 100 90 80 70 60 50 40 30 20 10
ppm

Figure S3B

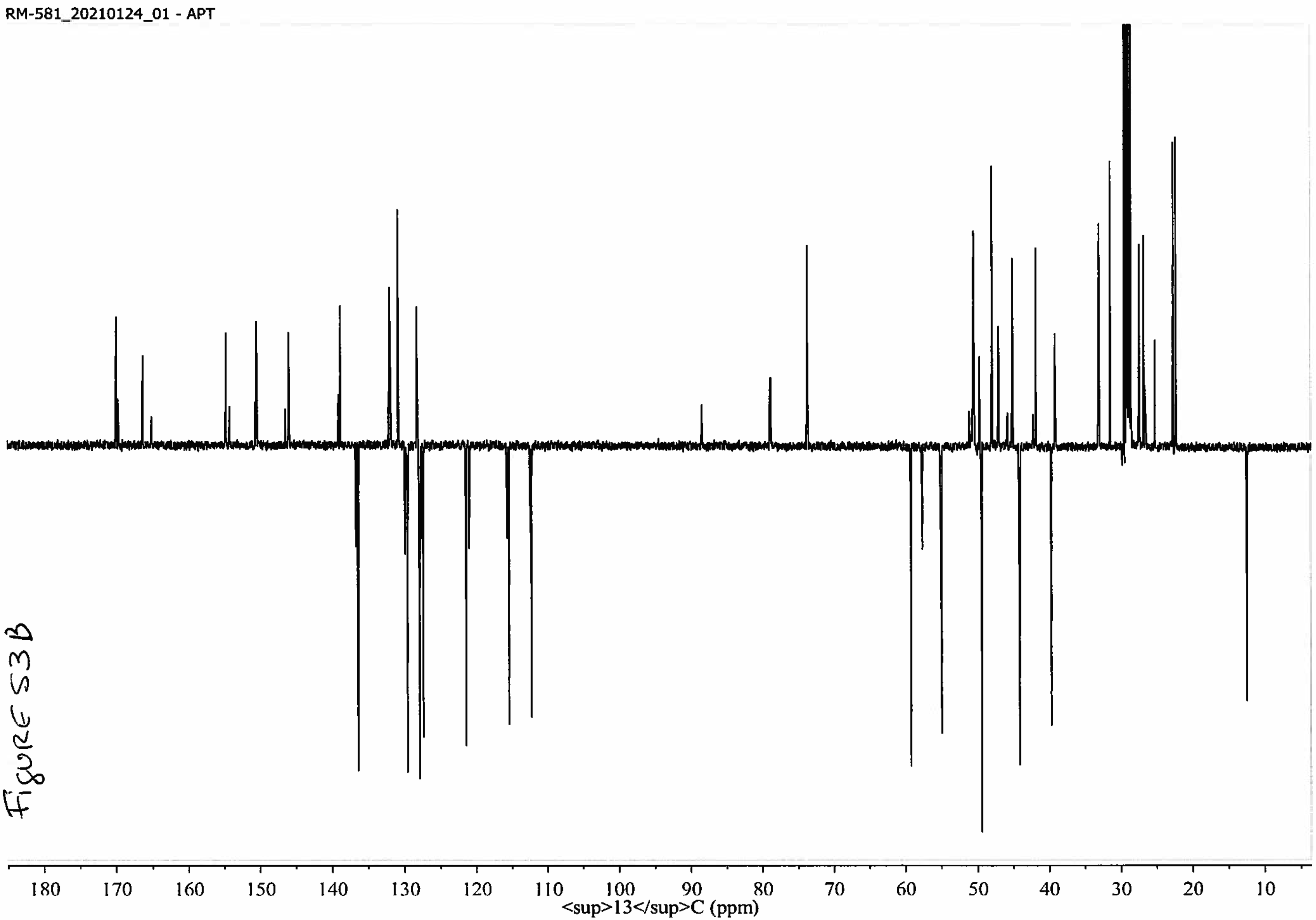
RM-581_20210124_01 - APT
180
170
160
150
140
130
120
110
100
90
80
70
60
50
40
30
20
10
¹³C (ppm)

FIGURE 54

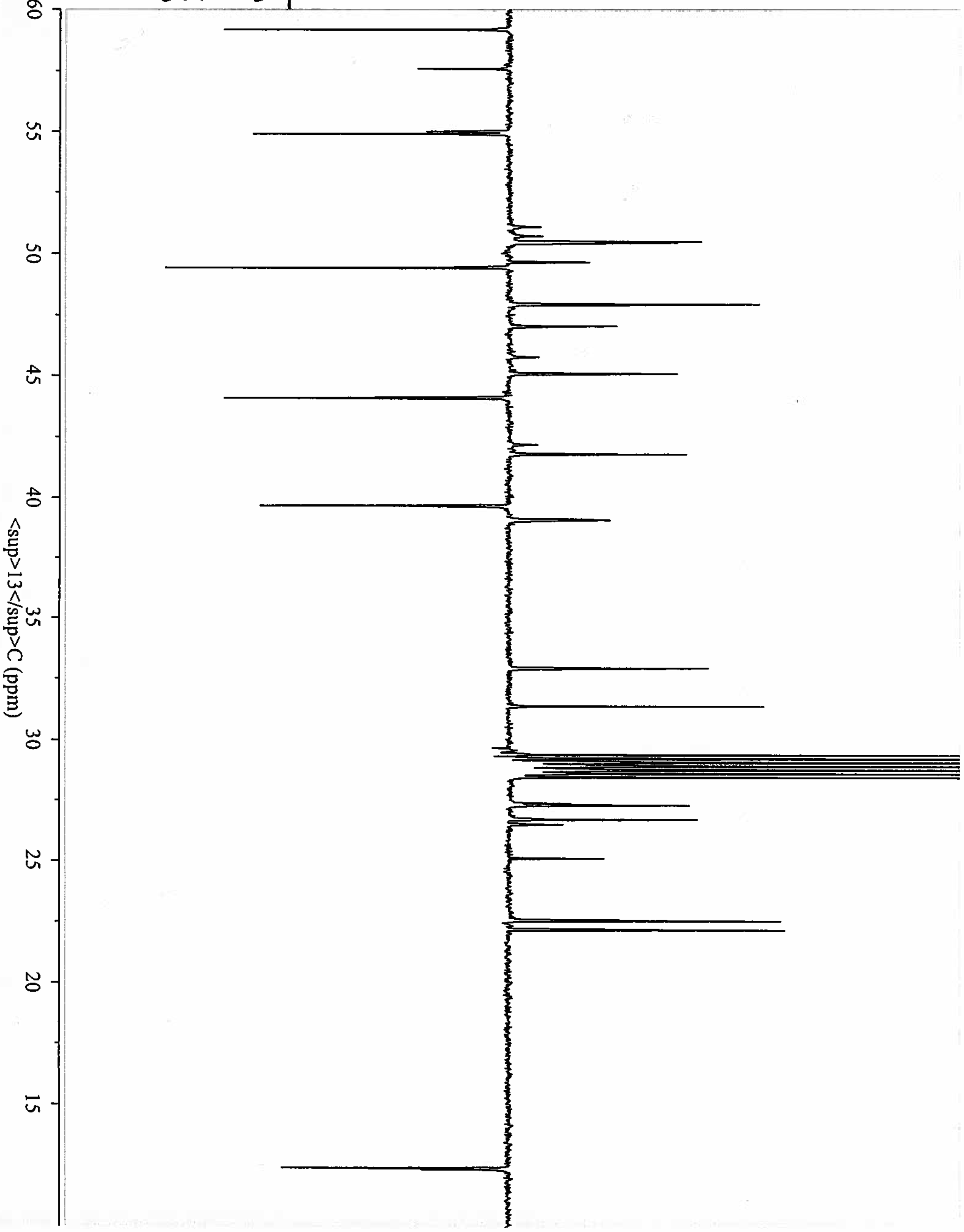

FIGURE 55

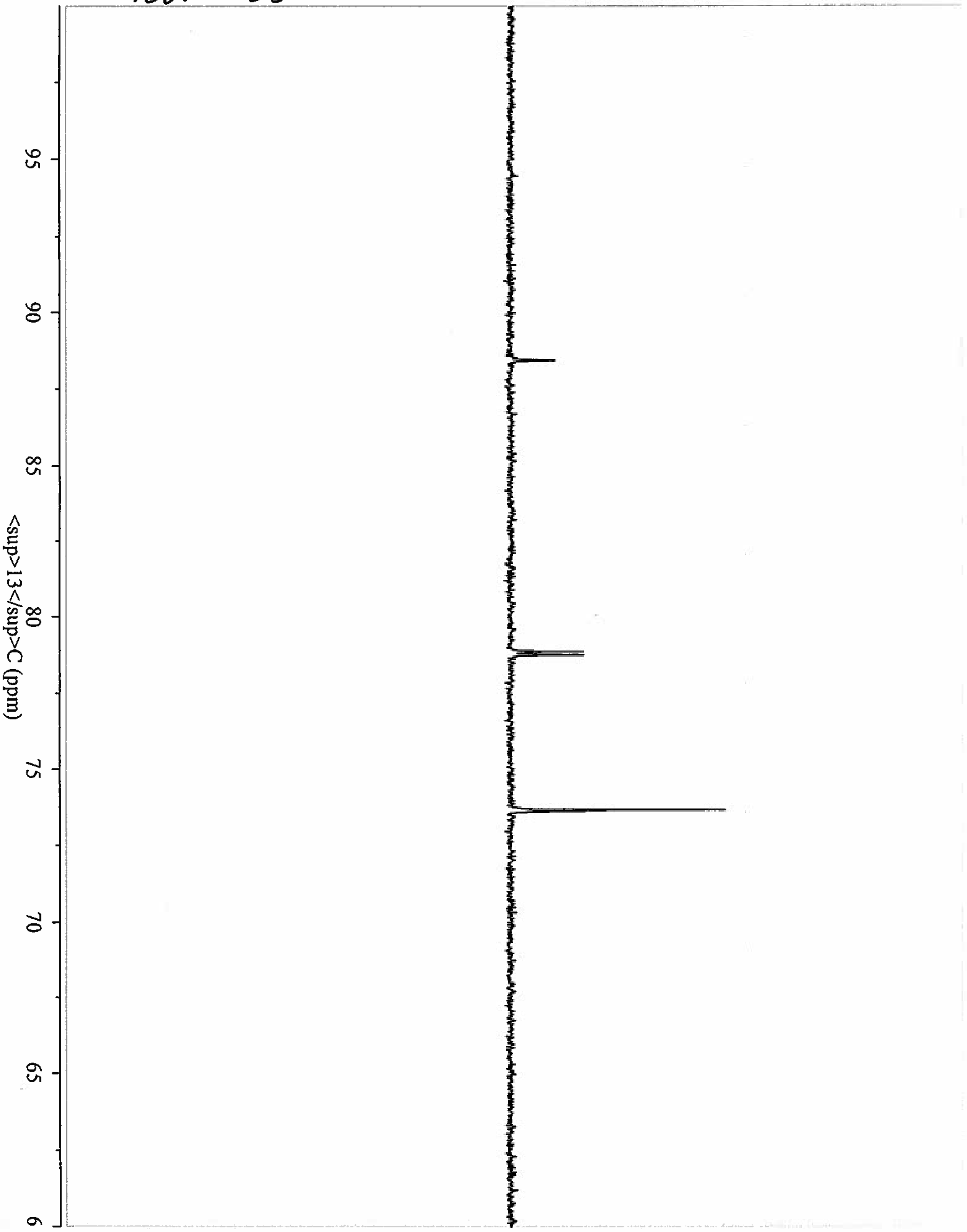

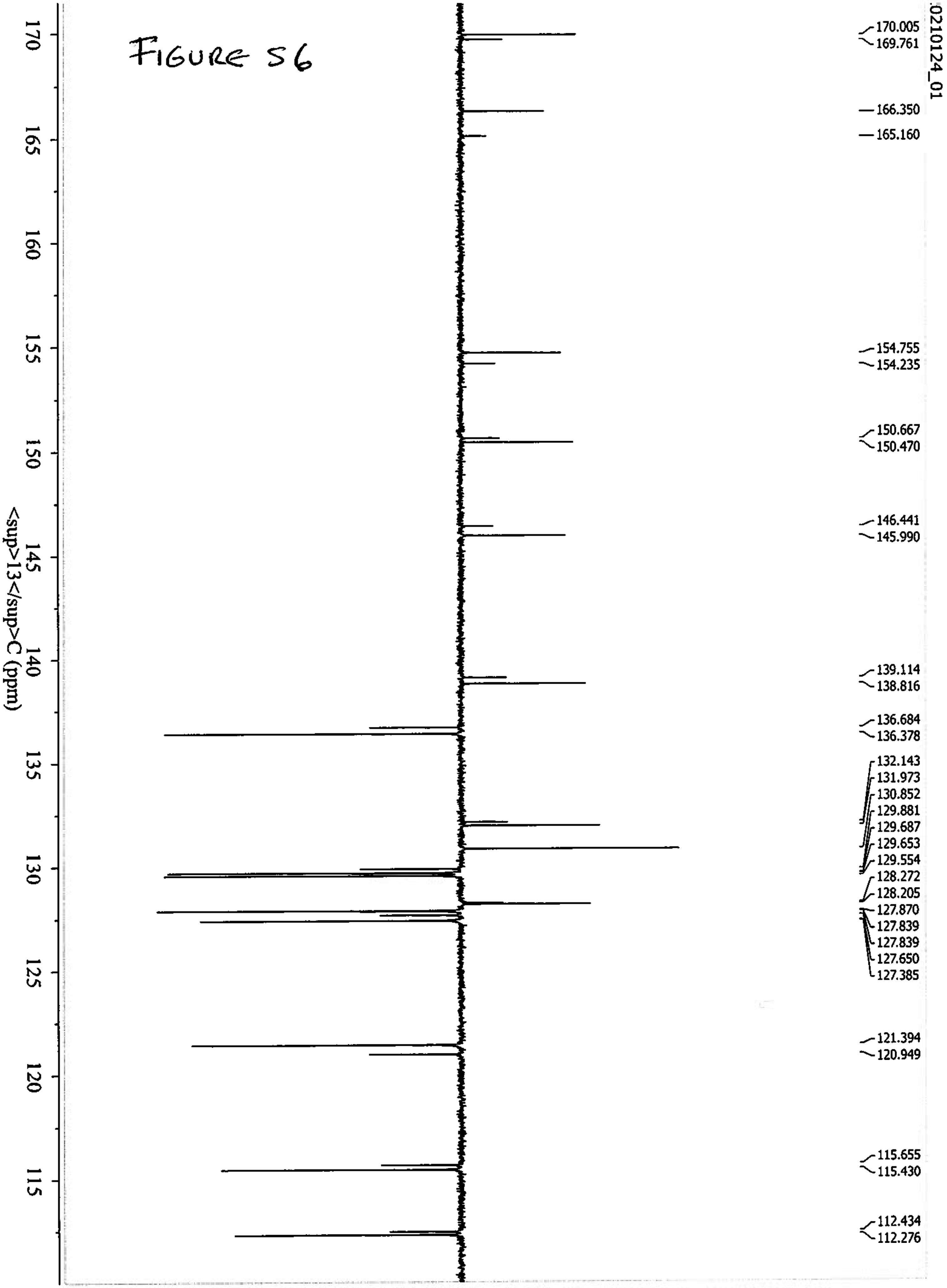
0210124_01
170.005
169.761
166.350
165.160
154.755
154.235
150.667
150.470
146.441
145.990
139.114
138.816
136.684
136.378
132.143
131.973
130.852
129.881
129.687
129.653
129.554
128.272
128.205
127.870
127.839
127.839
127.650
127.385
121.394
120.949
115.655
115.430
112.434
112.276
170
165
160
155
150
145
140
135
130
125
120
115
13C (ppm)

FIGURE S6

FIGURE S7

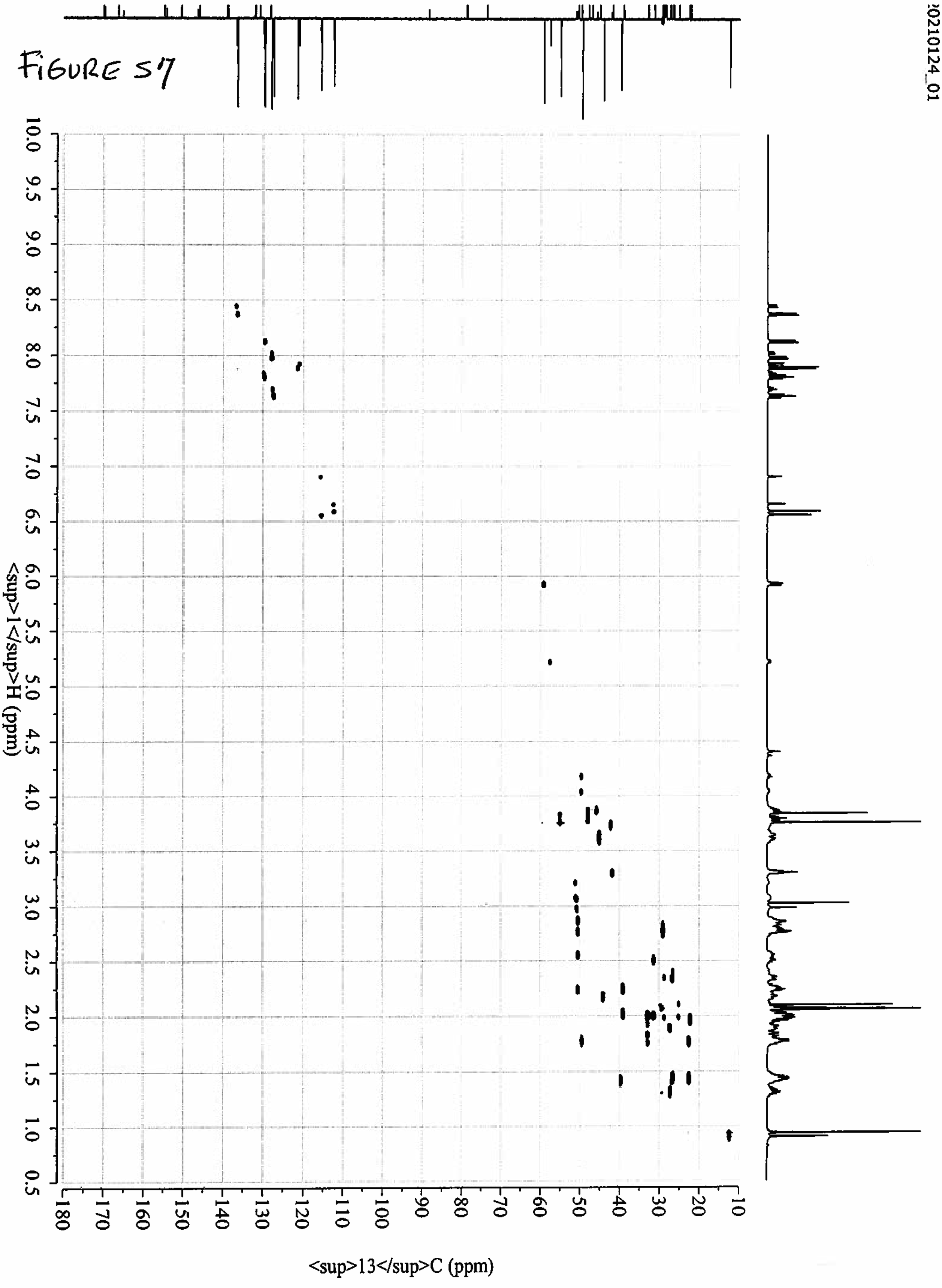

FIGURE 58

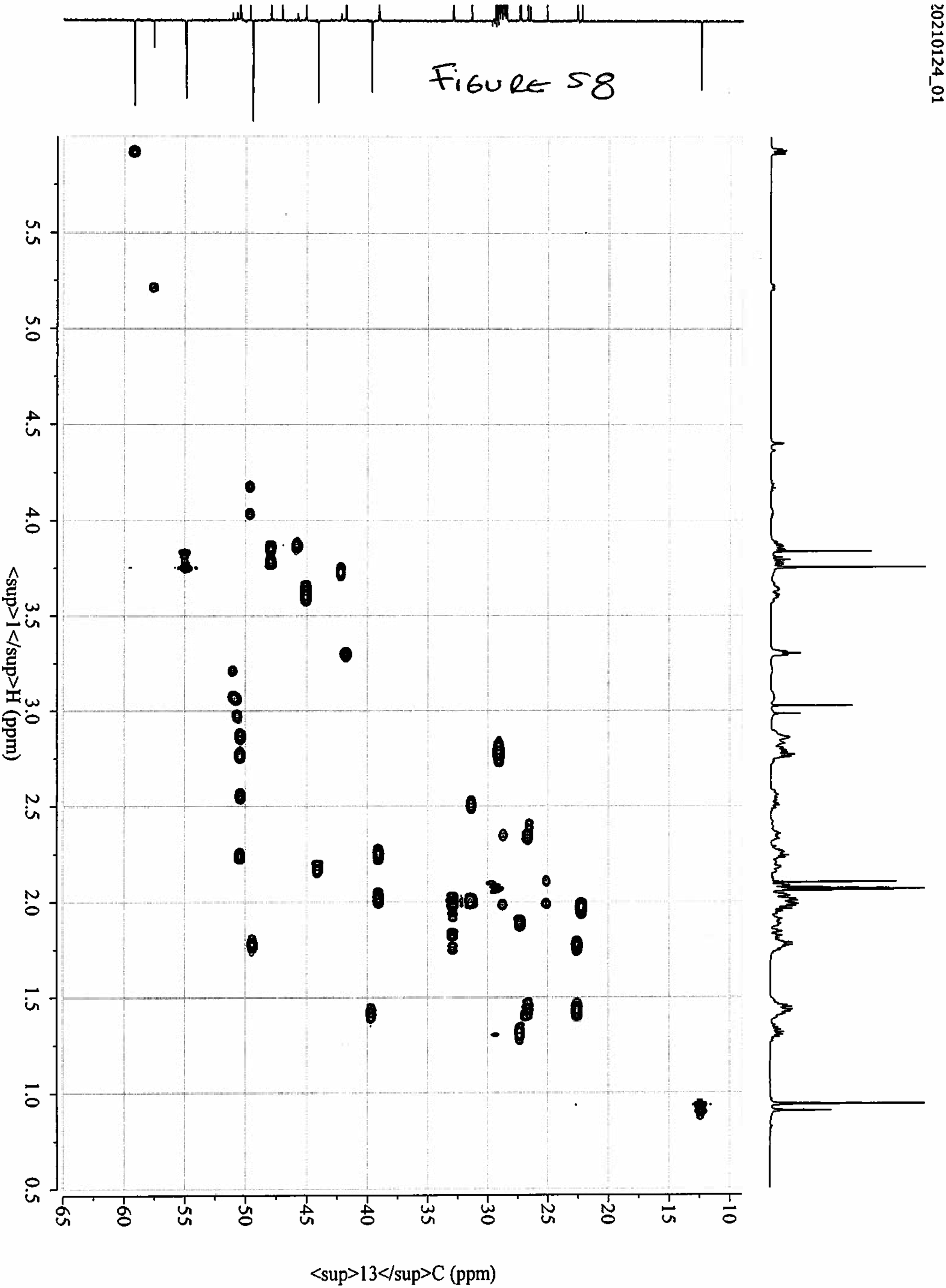
20210124_01
¹H (ppm)
¹³C (ppm)
65 60 55 50 45 40 35 30 25 20 15 10
5.5 5.0 4.5 4.0 3.5 3.0 2.5 2.0 1.5 1.0 0.5

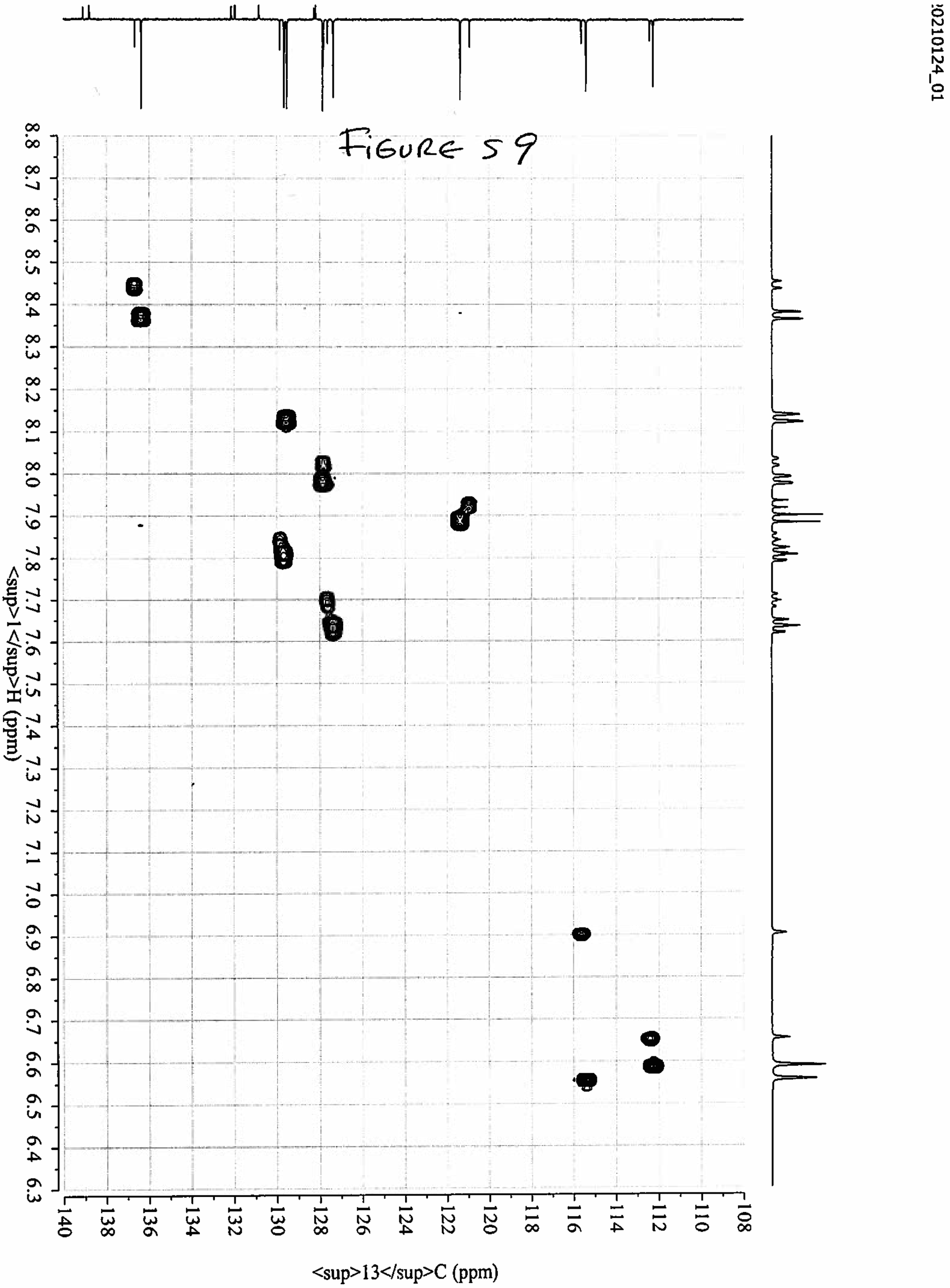

FIGURE S9

0210125_01 - HMBC

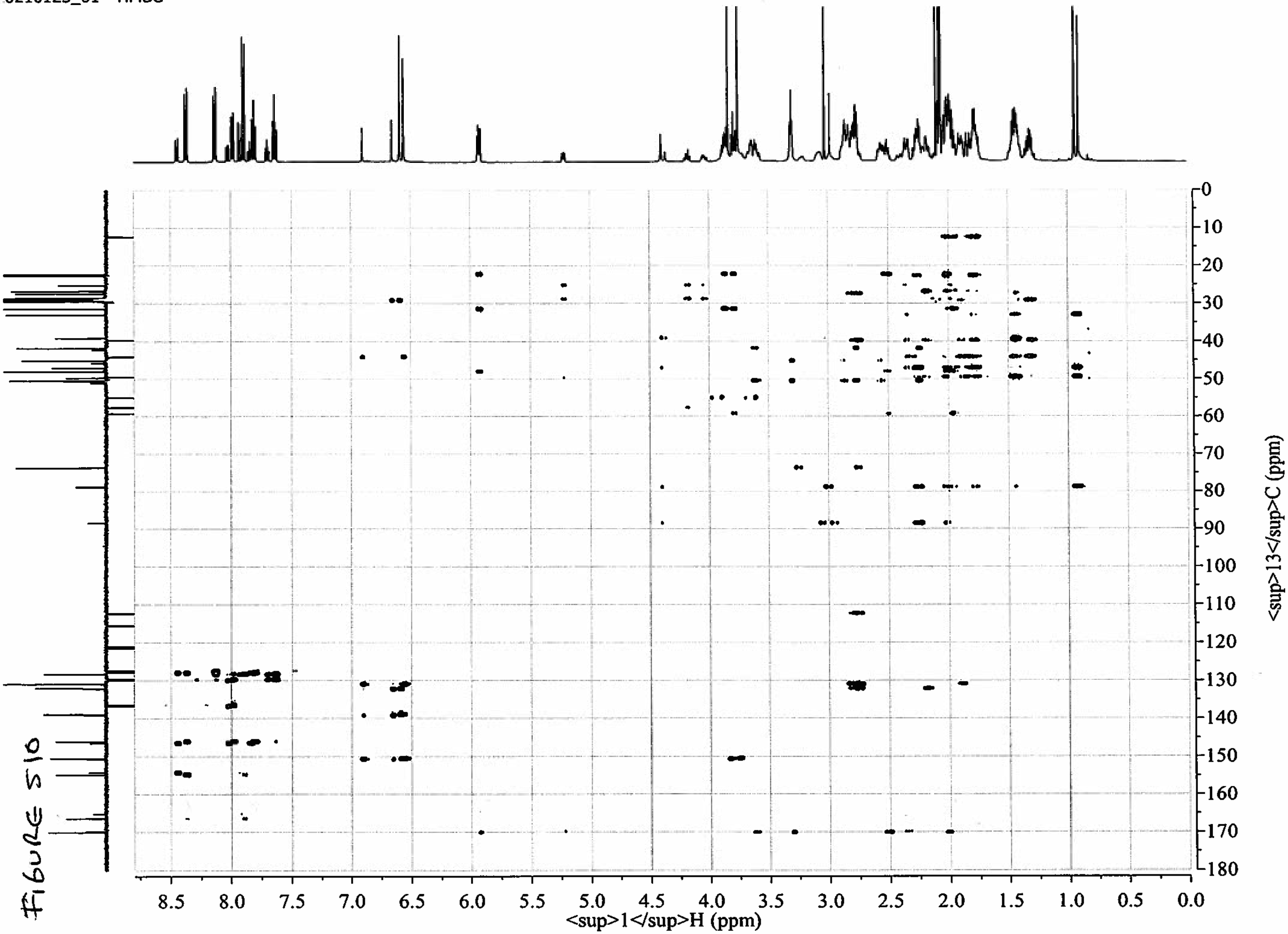

FIGURE S10

FIGURE S11

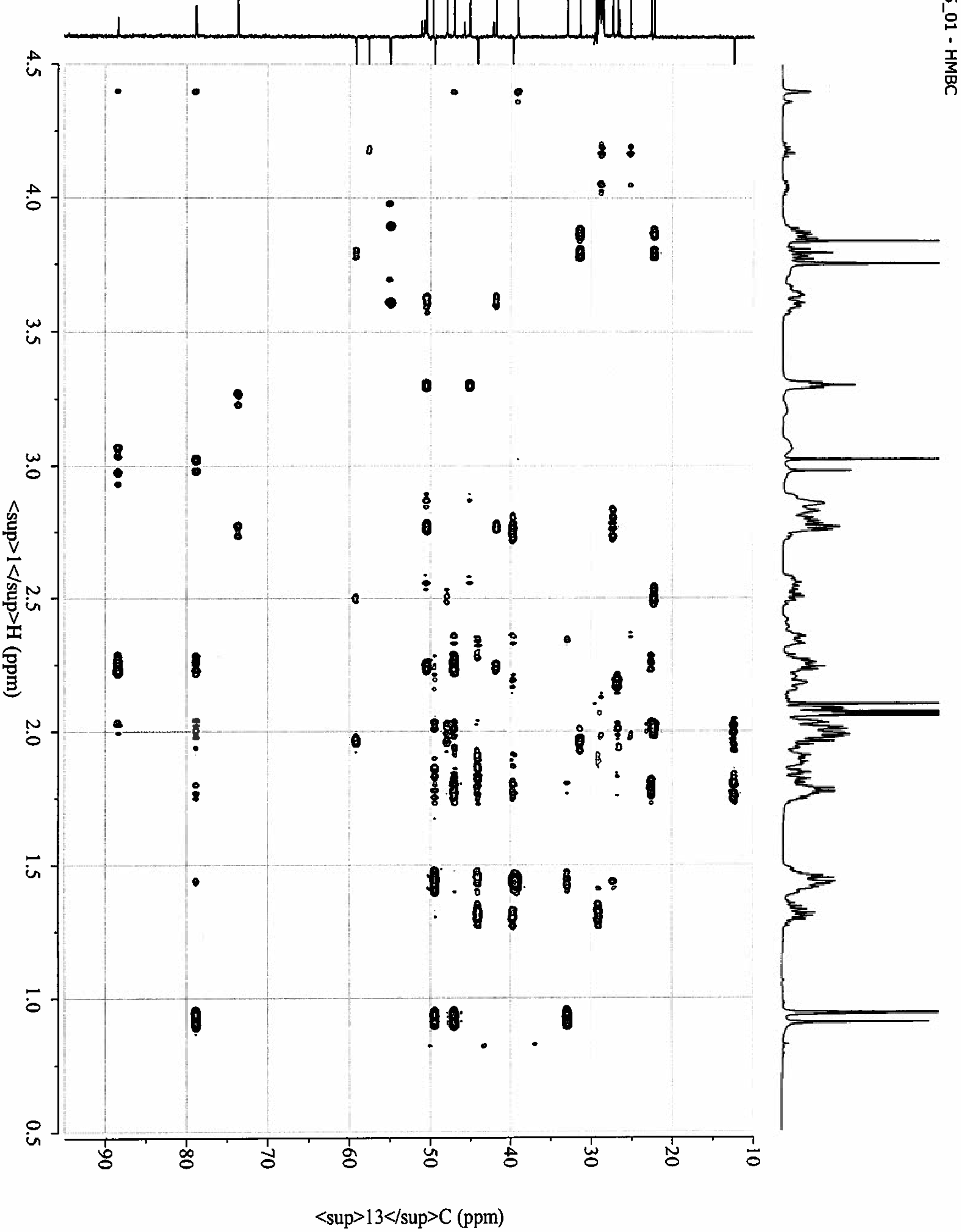
20210125_01 - HMBC
¹H (ppm)
¹³C (ppm)
4.5
4.0
3.5
3.0
2.5
2.0
1.5
1.0
0.5
90
80
70
60
50
40
30
20
10

FIGURE S12

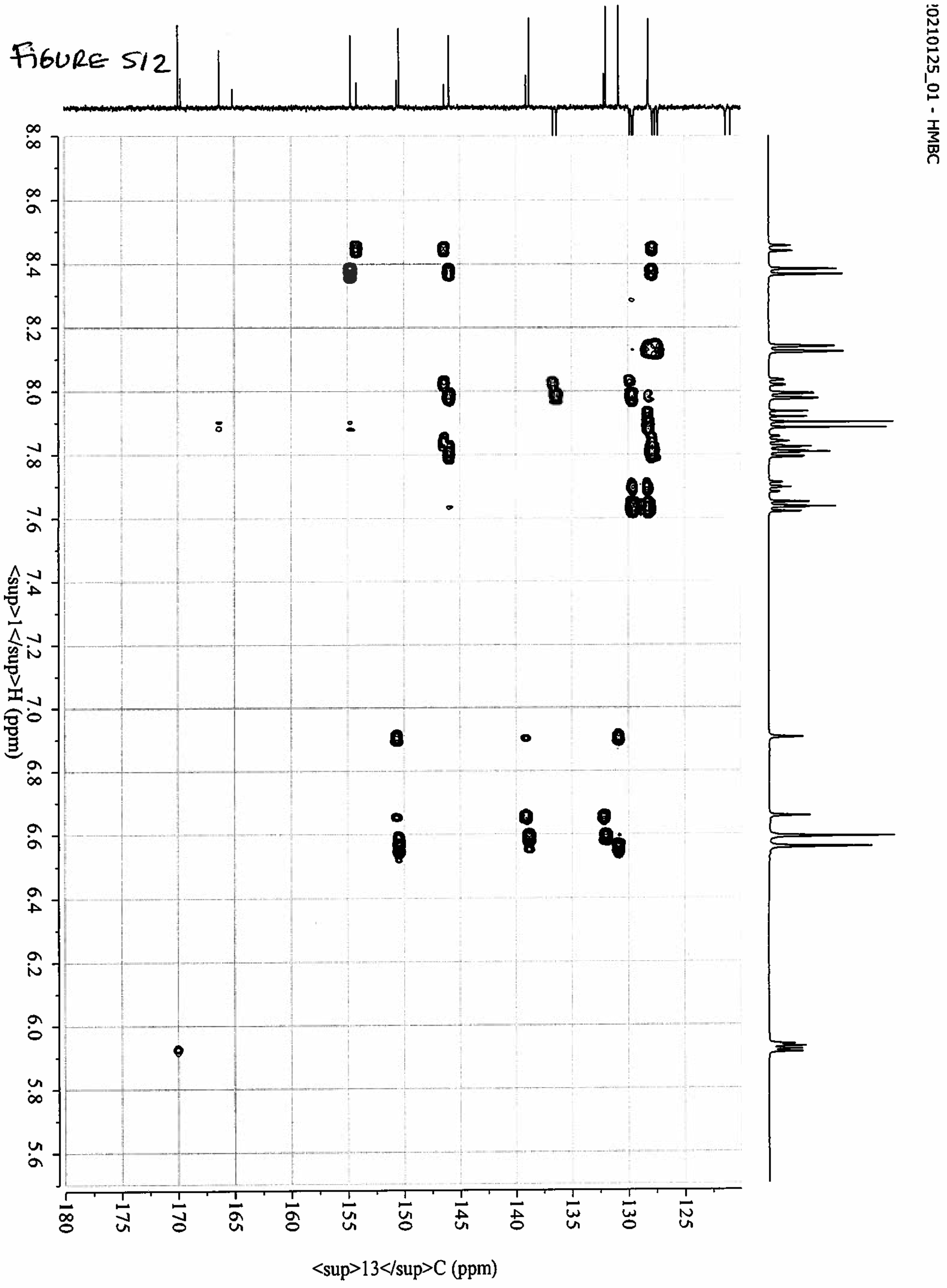

FIGURE S13

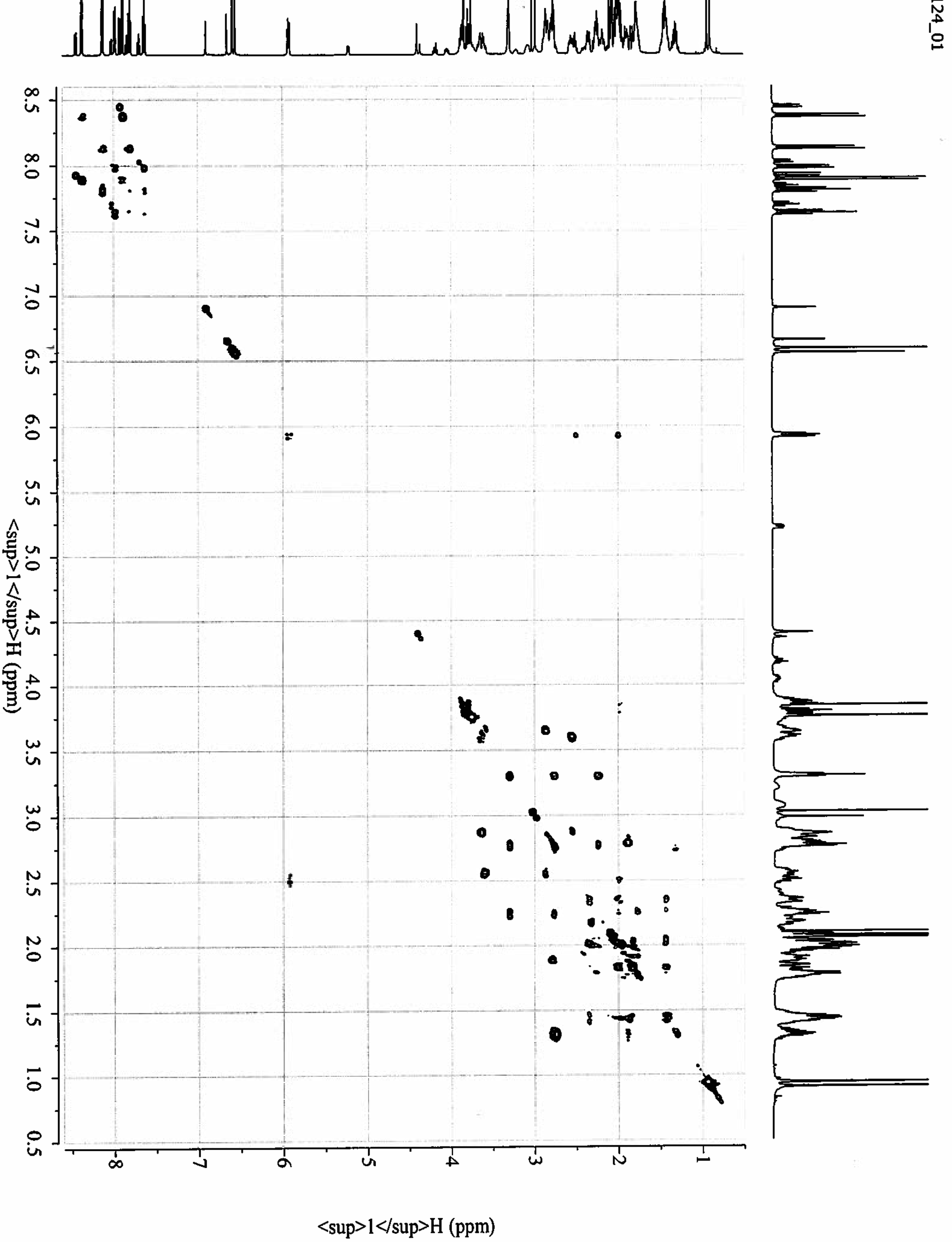

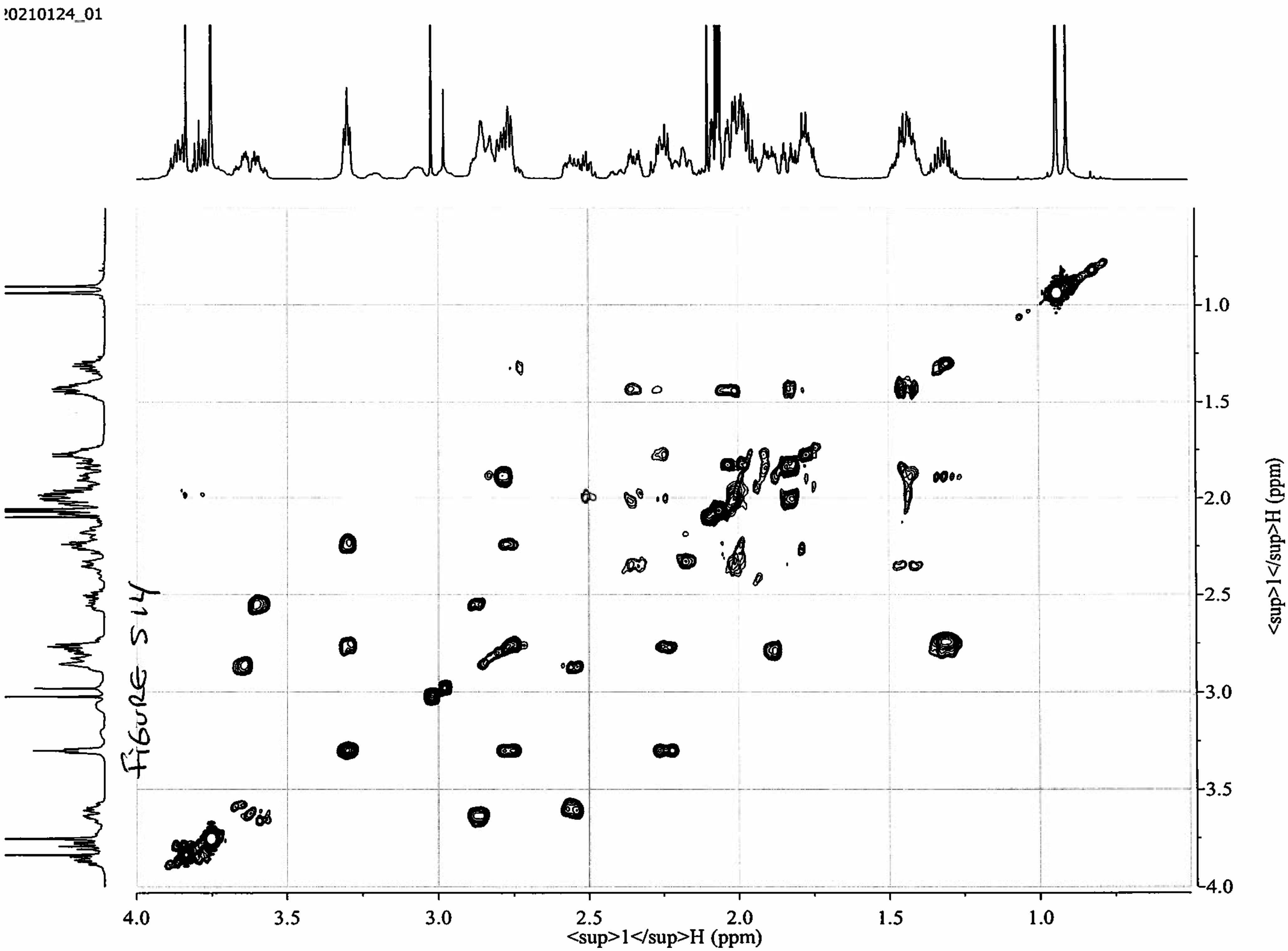
20210124_01
FIGURE S14
4.0
3.5
3.0
2.5
2.0
1.5
1.0
¹H (ppm)
¹H (ppm)

FIGURE S15

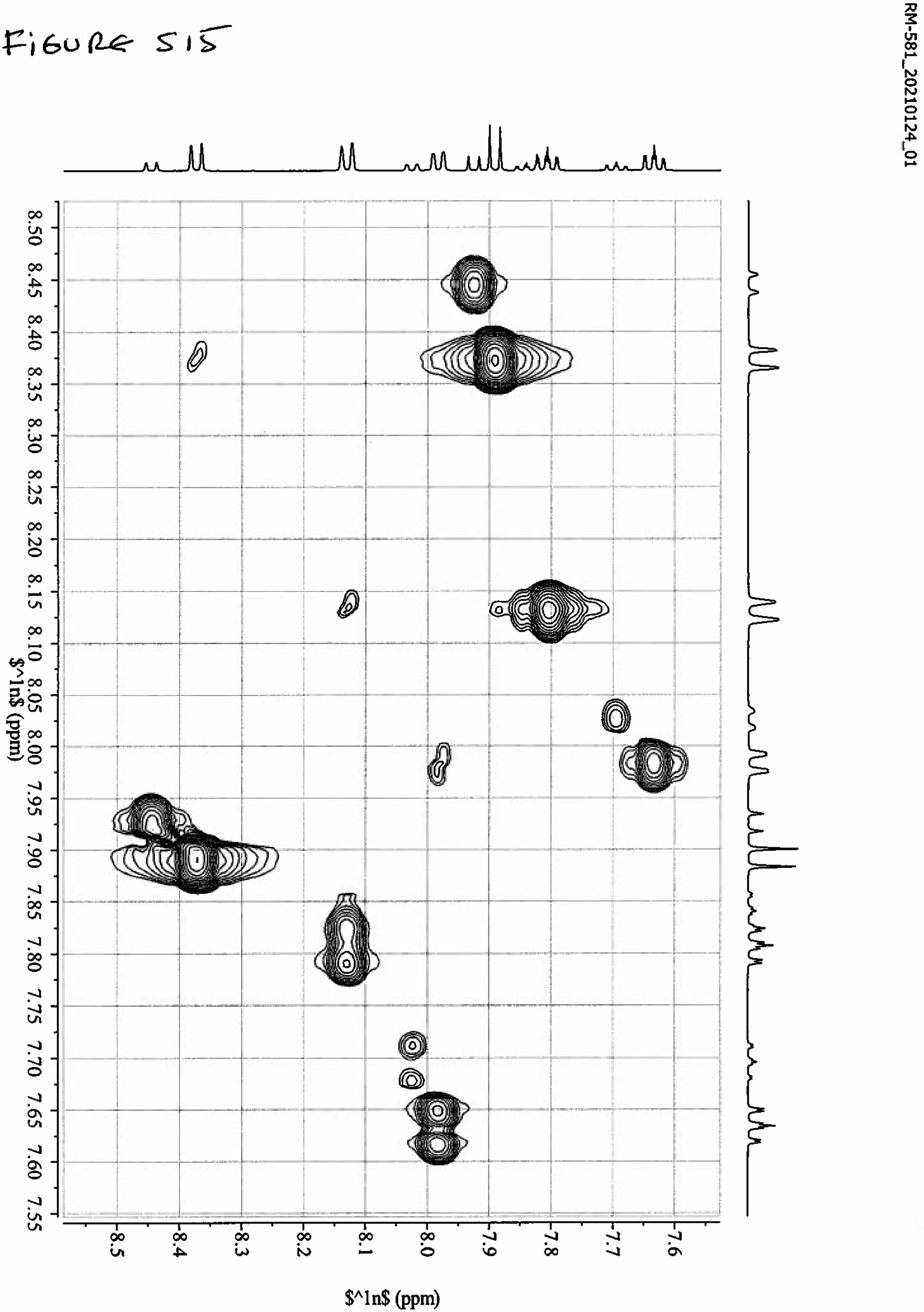

FIGURE S16

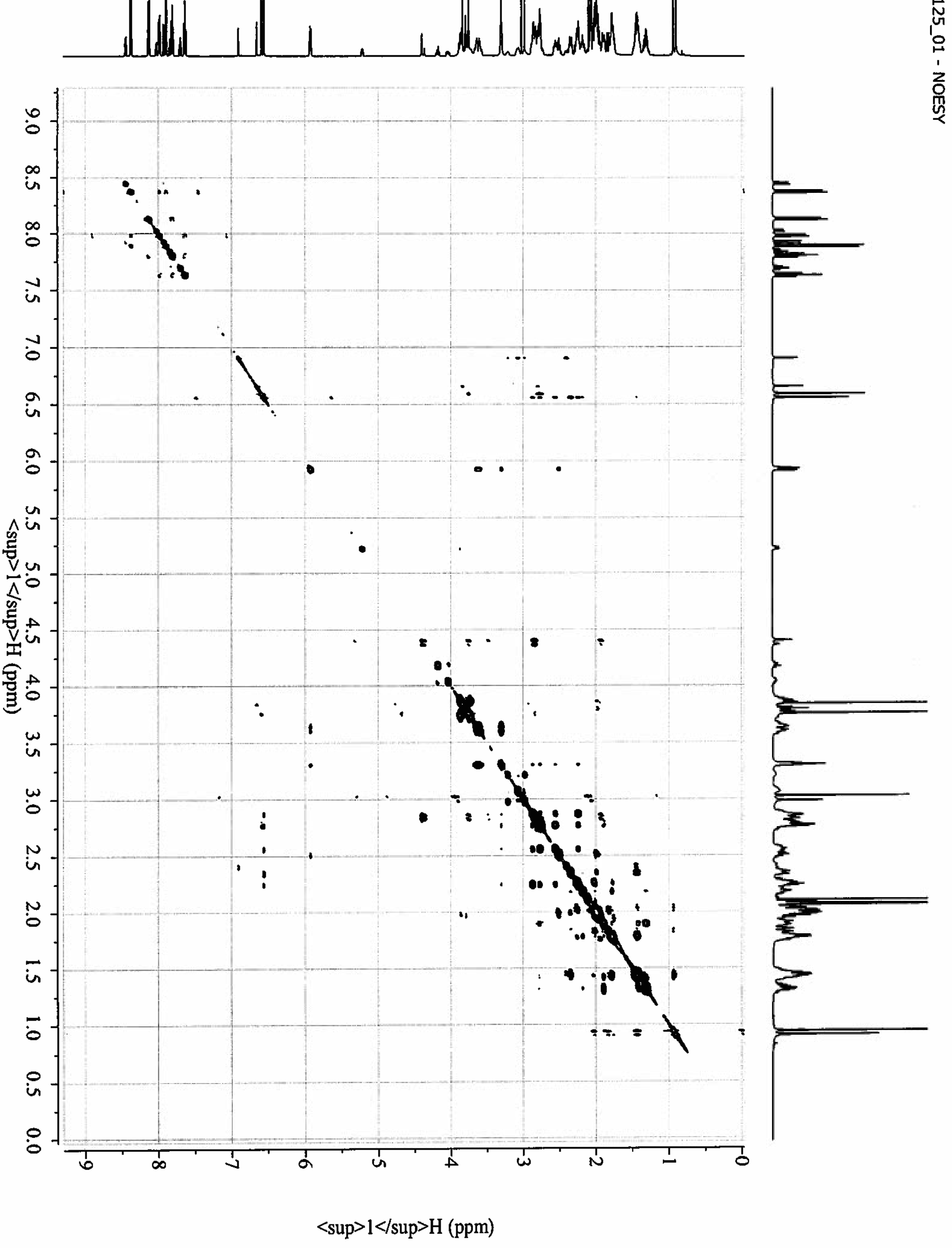

FIGURE S17

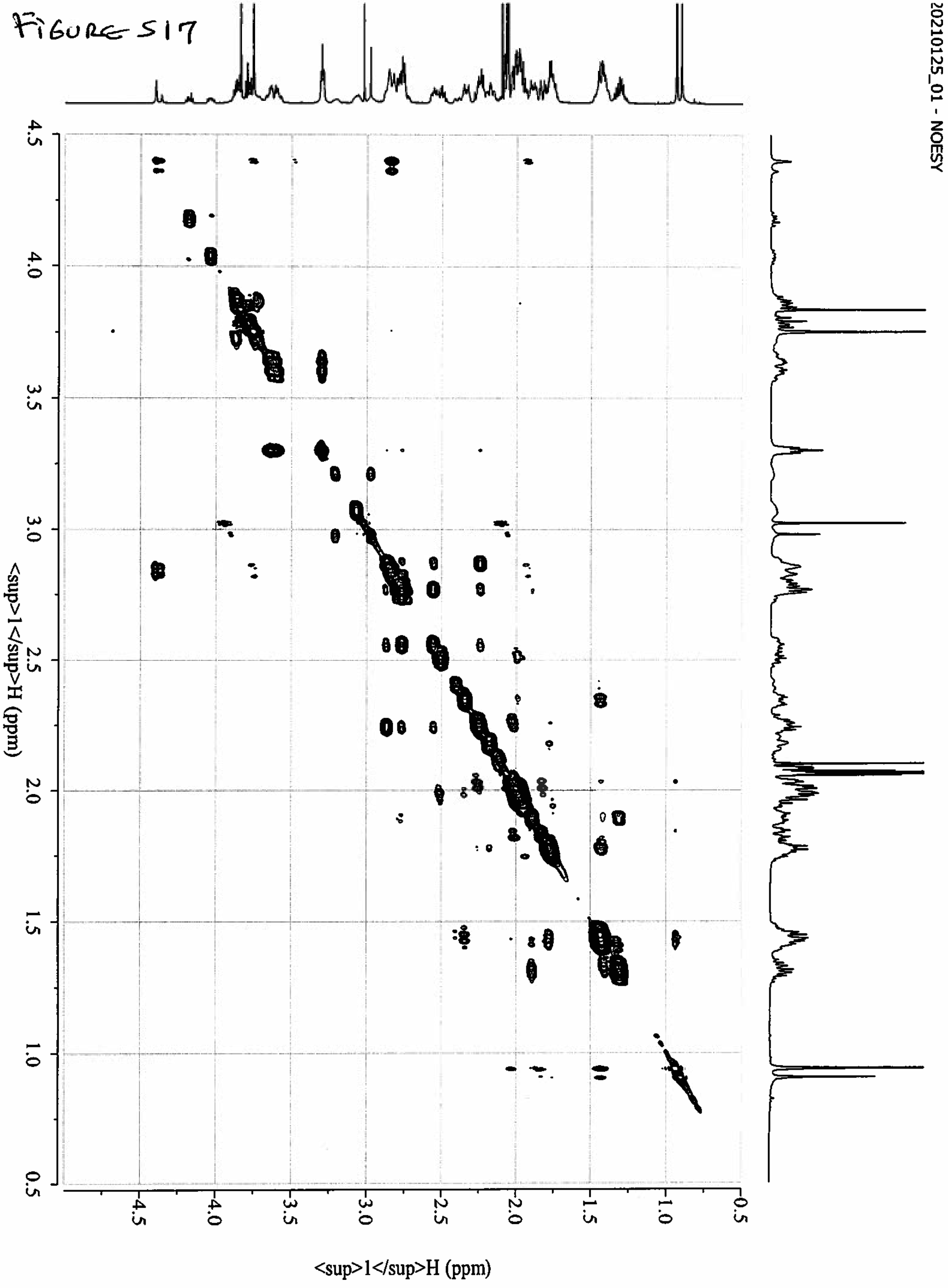

20210125_01 - NOESY
¹H (ppm)
¹H (ppm)
4.5
4.0
3.5
3.0
2.5
2.0
1.5
1.0
0.5

:0210125_01 - NOESY

FIGURE S18

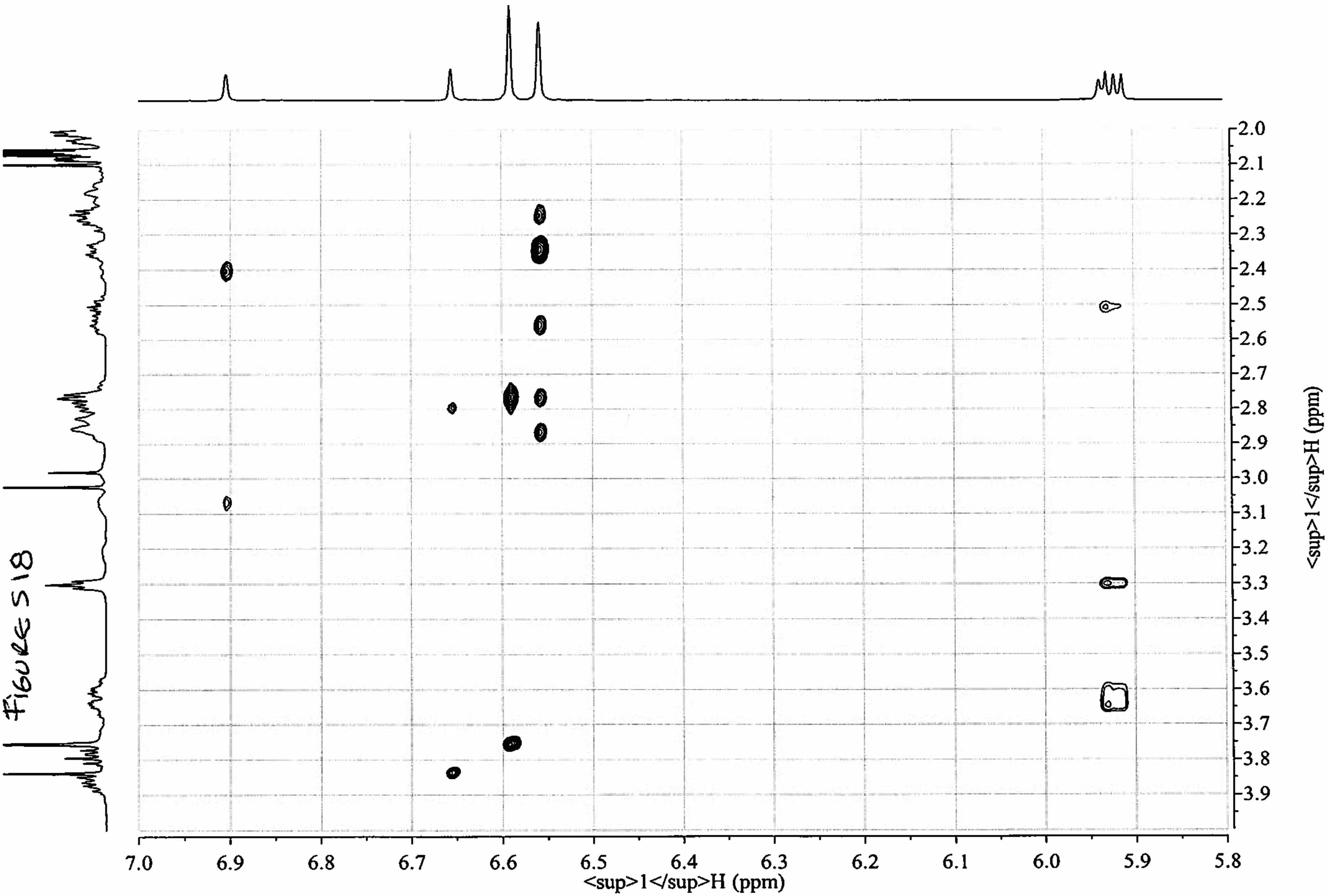

FIGURE S19

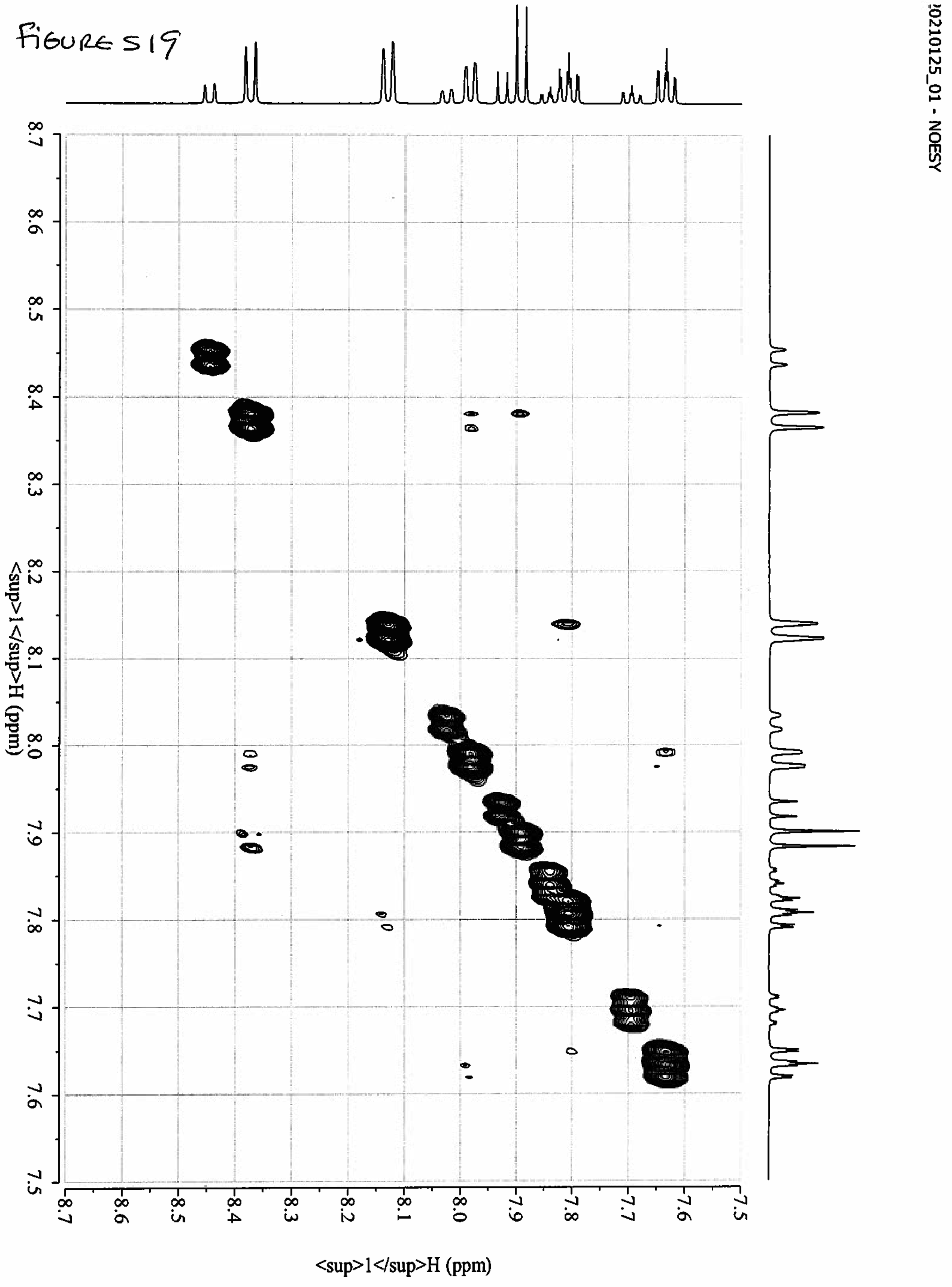

Supplement: Supplementary file 1 [file molecules-30-02441-s001.zip › molecules-3662941-supplementary.pdf]
